# A deep generative model for multi-view profiling of single-cell RNA-seq and ATAC-seq data

**DOI:** 10.1186/s13059-021-02595-6

**Published:** 2022-01-12

**Authors:** Gaoyang Li, Shaliu Fu, Shuguang Wang, Chenyu Zhu, Bin Duan, Chen Tang, Xiaohan Chen, Guohui Chuai, Ping Wang, Qi Liu

**Affiliations:** 1 Tongji University Cancer Center, Shanghai Tenth People’s Hospital of Tongji University, Tongji University, Shanghai, 200092 China; 2 Translational Medical Center for Stem Cell Therapy and Institute for Regenerative Medicine, Shanghai East Hospital, Bioinformatics Department, School of Life Sciences and Technology, Tongji University, Shanghai, China; 3 Shanghai Research Institute for Intelligent Autonomous Systems, Shanghai, China

## Abstract

Here, we present a multi-modal deep generative model, the single-cell Multi-View Profiler (scMVP), which is designed for handling sequencing data that simultaneously measure gene expression and chromatin accessibility in the same cell, including SNARE-seq, sci-CAR, Paired-seq, SHARE-seq, and Multiome from 10X Genomics. scMVP generates common latent representations for dimensionality reduction, cell clustering, and developmental trajectory inference and generates separate imputations for differential analysis and cis-regulatory element identification. scMVP can help mitigate data sparsity issues with imputation and accurately identify cell groups for different joint profiling techniques with common latent embedding, and we demonstrate its advantages on several realistic datasets.

**Supplementary Information:**

The online version contains supplementary material available at 10.1186/s13059-021-02595-6.

## Background

Cis-regulatory elements (CREs), which are bound by combinations of transcription factors, drive cell-type-specific and time-dependent regulation of gene expression. Genome-wide mapping of CREs and their activity patterns across cells and tissues can provide insights into the mechanisms of gene regulation. As CREs are mostly located in open chromatin regions, epigenomic sequencing technologies such as DNase-seq [1, 2] and ATAC-seq [3] have been developed to detect open chromatin regions and measure chromatin accessibility in tissues and cells. The advancement of single-cell technologies, such as scRNA-seq [4, 5] and scATAC-seq [6, 7], provides powerful tools to uncover complex and dynamic gene regulatory networks during tissue development across different cell types.

Recently, several joint profiling methods that allow simultaneous measurement of gene expression and chromatin accessibility in the same cell, such as SNARE-seq [8], sci-CAR [9], Paired-seq [10], and SHARE-seq [11] have provided accurate matching of chromatin accessibility landscape to gene expression profiles. Moreover, 10X Genomics recently developed a “multiome” approach. This new joint profiling platform would probably extend the rapid generation and wide application of single-cell multi-modal data. Although great advances have made in this field, these joint profiling technologies suffer from low throughput and data sparsity. These problems impede data interpretation and limit their application in data integration and downstream analysis like cell clustering and CRE identification. Currently, several analysis methods support data integration from different modalities 12–14 and CRE interaction analysis based on either scRNA-seq [12] or scATAC-seq [13, 14] data. However, these methods cannot address the obstacle of extreme data sparsity in joint profiling technologies and use only a fraction of differentially expressed genes and differentially accessible elements in CRE interaction analysis [9]. Also, previous integration algorithms cannot address divergence among the heterogeneous multi-omic data, as the discrete ATAC-seq data for hundred thousands of open chromatin regions and the continuous RNA-seq data for thousands of genes. To address these issues, several algorithms based on statistical framework [15, 16] or deep generative framework [17, 18] provided different approaches for comprehensive integration of both paired and unpaired single-cell datasets. More recently, Seurat released a beta version v4.0 for integrative multimodal analysis of joint modality single-cell datasets using weighted nearest neighbour (WNN) analysis [19], which is applied to 10X Genomics multiome datasets. Another work tested the application of multiple neural networks for integrative multimodal integration analysis, which used different joint strategies in different datasets [20], but lacked of available tools or code for real application to multi-modal datasets.

Deep generative models have been widely applied for modeling the high-dimension data, such as singe-cell sequencing data [17, 18]. Among those deep generative models, the variational autoencoder (VAE), which uses a recognition module as encoder and a generative module as decoder to learn the latent distribution of input data. The VAE model maximizes the similarity between generated data from decoder and input data while minimizing the Kullback-Leibeler divergence of the prior distribution of latent embedding and its true posterior distribution produced by the inference (encoder) network. The standard VAE model uses a multivariable Gaussian distribution as prior for the latent variables, which is hard to fit for sparse data with complex distribution. Replacing Gaussian distribution with Gaussian Mixture Model (GMM) as the prior has been applied in a recent developed method SCALE for unsupervised clustering and realistic samples generation for scATAC-seq datasets [14]. Recent tools as MultiVI [18] and Cobolt [17] utilize symmetric multimodal VAE model for joint modality single-cell dataset. However, for the multi-modal data integration, the encoder-produced latent embedding can capture the common semantic feature across modalities while decoder-generated data still preserve the modal-specific biological information, which require the similarity between integrated modalities. For joint profiling datasets with extreme data sparsity and random noise in either omic of dataset, the inconsistency of multi-omics joint embedding will largely confuse the biological variation in cell latent embedding and exceedingly smooth the generated data from continuous distribution of generative model, impeding the explanation and downstream application of joint latent embedding. In addition, self-attention-based embedding models, such as Transformer and BERT, show high performance on extreme sparse NLP tasks [21] and sequence or structured tasks like protein-structured prediction [22], indicating their potential in capturing the weak correlation from high-dimensional high-sparsity biological data.

Here, we propose a non-symmetric deep generative model, the single-cell Multi-View Profiler (scMVP), which is designed for comprehensive handling sequencing data that simultaneously measure gene expression and chromatin accessibility in the same cell, including SNARE-seq [8], sci-CAR [9], Paired-seq [10], SHARE-seq [11], and 10X Multiome. scMVP automatically learns the common latent representation for scRNA-seq and scATAC-seq data through a clustering consistency-constrained multi-view variational auto-encoder model (VAE), and imputes each single layer data from the common latent embedding of the multi-omic data through layer-specific data generation process, including transformer’s self-attention-based scATAC generation channel and mask attention-based scRNA generating channel. scMVP is designed specifically to address the two main challenges in joint profiling of scRNA-seq and scATAC-seq, i.e., (1) how to overcome the difficulties in processing a highly sparse data matrix, as the sequencing data throughput of the joint profiling methods is only one-tenth to one-fifth the throughput of single modality scRNA-seq or scATAC-seq data; (2) how to jointly utilize two omic data for downstream single-cell analyses, such as cell denoising, cell clustering, cellular trajectory inference, and CRE prediction rather than conventional independent analysis of scRNA and scATAC followed by integration or anchoring the two omics data between similar cell clusters. Compared to other tools which utilize neural networks for embedding scRNA-seq datasets [23–27] and multi-modal datasets [15–18], scMVP provides an efficient deep generation model for joint profiling of multiple omic measurements of the same single-cell and enables simultaneous multi-modal analysis of data normalization, clustering, joint embedding, visualization, trajectory inference, and CRE prediction for joint profiling sequencing data.

## Results

### The *scMVP* model

**Fig. 1:**
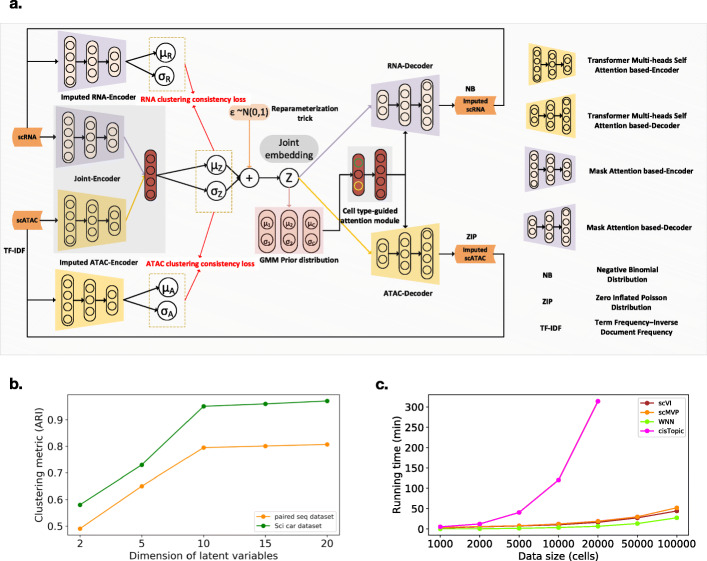
Overview of the scMVP framework. **a** Given the scRNA-seq genes expression counts and TF-IDF transformed scATAC-seq chromatin accessibility peaks profile of each cell as input, scMVP learns the optimal joint embedding for downstream analysis with a multi-view deep generative model. Two independent channels of attention-based networks are utilized to the backbone of the encoder model to adapt inputs of the different modalities, including canonical mask attention subnetwork for scRNA and transformer derived self-attention for TF-IDF transformed scATAC, and then joint together to derive the posterior distribution parameters of common latent embedding *z* following a Gaussian mixture model prior. Next, the imputed scRNA and scATAC profiles are reconstructed by an attention based two-channels decoder network, which shares similar network structure with the encoder network. And an auxiliary attention module with input of cluster probability of common latent embedding *z* (denoted as *p*(*c*| *z*)) in the prior distribution is utilized to weight each decoder channel of the imputed scRNA and scATAC profile. Here, the imputed RNA and ATAC are produced by the mean value of Gamma distribution for scRNA data and the Poisson distribution for scATAC data, respectively. To guarantee the embedding consistency between the original and imputed data, two single-channel encoders are used to embed the imputed RNA and ATAC separately to minimize the KL divergence between common latent embedding *z* and each imputed embedding. **b** ARI metrics of clustering accuracy along with the varying of latent embedding dimensions in a range from 2 to 20. **c** Running times for training models on the resampling SHARE-seq cell line datasets with a set of 8000 genes and 23,000 peaks. scMVP, scVI, WNN, and cisTopic are tested on a server with one 10-core Intel Xeon E5-2680 with 32 GB RAM and one NVIDIA 1080TI GPU with 11 GB RAM

To fully utilize the joint profiling data from the same cell, we developed scMVP, which integrated scRNA and scATAC data into a common low-dimensional latent space for cell embedding, clustering, and imputation (Fig. 1a).

The basic idea of scMVP is to introduce a Gaussian mixture model (GMM) prior to derive the common latent embedding by maximizing the likelihood of the joint generation probability of the multi-omic data, which is implemented as a multi-modal asymmetric GMM-VAE model with two extra clustering consistency modules to align each imputed omics and preserve the common semantic information, and used to impute missing data, cluster cell groups, assemble multiple modalities, and construct a developmental lineage.

First, scMVP takes raw count of scRNA-seq and term frequency–inverse document frequency (TF-IDF) transformed scATAC-seq as input [28]. To auto-learn a common latent distribution of the joint scRNA-seq and scATAC-seq profiling, scMVP utilizes GMM as the prior distribution of latent embedding *z* for the multi-view VAE model, that is, the observed scRNA gene expression *x* and TF-IDF transformed scATAC chromatin accessibility *y* in each cell modeled as a sample drawn from a negative binomial (NB) distribution *p*(*x*| *z*, *c*) and a zero-inflated Poisson (ZIP) distribution *p*(*y*| *z*, *c*), conditioned on the common latent embedding *z* and cell type *c*, one of predefined *K* components of GMM. scMVP uses a two-channel Decoder neural network transforming the common latent embedding z into the parameters of NB and ZIP distribution, with a cell type *c* guided attention module to capture the potential correlation between the scRNA and scATAC data within same cell (see Fig 1a and method). Then, the generated scRNA and scATAC data are denoised and imputed by the mean of the corresponding output distribution, respectively, while the embedded common latent code *z* can be used for a series of downstream analysis, e.g., visualization, trajectory analysis, and which is inferenced through a variational process by maximizing the variational evidence lower bound (ELBO), that is, ${\mathcal{L}}_{elbo}\left(x,y\right)={E}_{q\left(z,c|x,y\right)}\left[\log \frac{p\left(x,y,z,c\right)}{q\left(z,c|x,y\right)}\right].$ scMVP estimates the distribution parameters of the *q*(*z*, *c*| *x*, *y*) according to another joint Encoder neural network, e.g., the mean *μ*_*z*_ and variance *σ*_*z*_ for *z* = *μ*_*c*_ + *σ*_*c*_*I*, *I*~*N*(0, 1) using a reparameterization trick for the gradient back-propagation. To better capture the feature correlations intra-omic and extract the biological intrinsic semantic embedding of inter-omics, we introduce the multi-heads self-attention-based transformer encoder and decoder modules for ATAC sub-network branch and mask attention-based encoder and decoder modules for RNA sub-network branch (see Fig. 1a and method). scMVP introduces the multi-heads self-attention module to capture the local long-distance correlation from sparse and high-dimension scATAC profile of joint dataset, and the mask attention to focus on the local semantic region of cells. Next, scMVP uses a cycle-GAN like auxiliary network module for consistency of latent embedding distribution between imputed and raw joint profiling data, and this auxiliary network module will enforce the latent embedding contain the common biological semantics as cell clusters across modalities rather than a simple alignment in canonical VAE and perverse the reversibility and uniqueness of each imputed omics (Fig. 1a and methods). Finally, the proposed model is trained using a back-propagation algorithm in a mini-batch way and generates latent embedding, scRNA-seq imputation, and scATAC-seq imputation simultaneously as output. The details of scMVP design can be found in the “Methods” section.

We further explored the optimal variable for latent dimensions. We constructed two datasets with well labelled cells from Paired-seq and sci-CAR cell line datasets and evaluated the clustering accuracy using adjusted Rand Index (ARI) metric depending on different dimensions of latent embedding. The higher ARI score indicates higher clustering accuracy, and the ARI score equals to 1 when the cluster is exactly matched to the reference standards. scMVP showed best performance with 10 dimensions of latent embedding, which is set as default size for latent embedding (Fig. 1b).

### *scMVP* model evaluation

We evaluated scMVP along with a set of benchmark methods on several single-cell joint profiling datasets with variable biological or technological characteristics [8–11]. We first tested the scalability of scMVP model on different joint profiling datasets. To estimate the time and memory consumption in the training step, we randomly sampled a range of 1000 to 100,000 cells from the 67,418 cells of SHARE-seq GM12878 cell line dataset and filtered dataset to 8000 genes and 23,000 peaks with highest expression, and tested the datasets of scRNA-seq in scVI, scATAC-seq in cisTopic and both in scMVP and Seurat v4 WNN. scMVP took the 752 MB for 1000 cells and 8.5 GB for 100,000 cells, which is similar with scVI, cisTopic, and WNN testing on 100,000 cells. Benefit from the GPU parallel computing technique and stochastic optimization in a minibatch way in the neural network model training, deep models as scMVP and scVI took similar training time with the general machine learning method WNN, which used less than 1 h for 100,000 cells dataset, while cisTopic based on Monte Carlo sampling model took more than 5 h for 20,000 cells dataset (Fig. 1c). To evaluate the capacity of scMVP for batch correction, we used the SHARE-seq GM12878 cell line dataset [11] containing 2 replicates of 2973 cells and 8803 cells, which showed batches between replicates in both scRNA-seq and scATAC-seq datasets (Additional file 1: Fig. S1a). scMVP successfully removed the batch from replicates without the label of batches (Additional file 1: Fig. S1a). In addition, convergence analysis showed scMVP reaching stable loss within 30 epochs for the SHARE-seq dataset, which would also be helpful to reduce the model training time (Additional file 1: Fig. S1b).

**Fig. 2:**
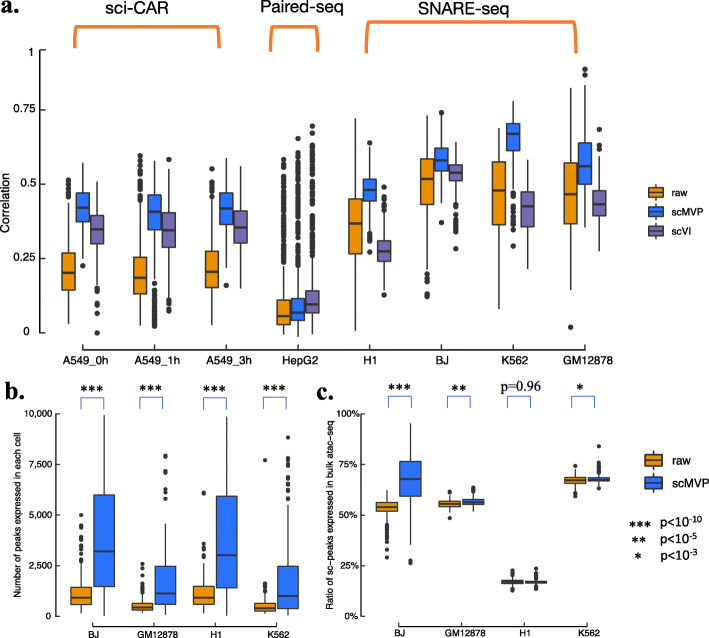
scMVP mitigates data sparsity in joint profiling datasets. **a** Correlation between original and imputed gene expression of each cell from scRNA of joint profiling datasets and gene expression in corresponding bulk RNA-seq dataset. A549 cell lines treated with DEX for 0h (ENCSR632DQP), 1h (ENCSR656FIH), 3h (ENCSR624RID) in sci-CAR dataset, HepG2 cell line (ENCSR058OSL) in Paired-seq dataset and H1 (ENCSR670WQY), BJ (ENCSR000COP), K562 (ENCSR530NHO), and GM12878 (ENCSR000CPO) cell lines in SNARE-seq dataset were used for benchmark. **b** Number of bulk ATAC peaks identified by raw and imputed scATAC in each cell. **c** Ratio of raw and imputed scATAC peaks identified in bulk ATAC peaks. DNase-seq signal files for H1 (ENCSR000EMU) and BJ (ENCSR000EME), and ATAC-seq signal files for K562 (ENCSR868FGK) and GM12878 (ENCSR095QNB) were used for benchmark

Next, we evaluated whether imputation from generative models such as scMVP and scVI can help mitigate data sparsity issue in joint profiling dataset. We first evaluated the ability to accurately capture real gene expression profiles by comparing imputed and real scRNA-seq profile of each cell type to gene expression in bulk cell line datasets of corresponding cell type. For each cell type, we used the correlation between the gene expression in every cell and the gene expression in bulk cell line RNA-seq, as higher the correlation of all genes in each cell from scRNA-seq indicating better capture of real gene expression of bulk RNA-seq in distinct cell type. We found scMVP showed higher imputation correlation than scVI and raw scRNA count in A549 cells treated with DEX for 0h, 1h, and 3h from sci-CAR dataset and four cell types SNARE-seq dataset (Fig. 2a). For HepG2 cell from Paired-seq scRNA-seq imputation of scMVP and scVI were consistently better than raw scRNA-seq count, indicating the improvement of scRNA-seq imputation for three joint profiling techniques.

We further evaluated imputation of scATAC-seq from scMVP by comparing peaks identified in each cell to bulk ATAC-seq or bulk DNase-seq signal in corresponding cell line. Compared to raw scATAC-seq profile, scMVP scATAC imputation captured more peaks than raw scATAC-seq (*p* value < 10^-10^), with median of 4114, 3778, 1017, and 1251 imputed peaks versus 918, 922, 404, and 442 raw peaks in BJ, H1, K562, and GM12878 cell lines (Fig. 2b). As scMVP imputed more scATAC-seq peaks in each cell than raw scATAC-seq profile, the ratio imputed peaks identified in bulk DNase-seq (H1, BJ) or bulk ATAC-seq (K562, GM12878) were higher in BJ, GM12878, and K562 cells and similar in H1 cells to the ratio of raw peaks in bulk dataset (Fig. 2c), which indicates enhancement of true ATAC-seq signal and mitigation of data sparsity for scATAC-seq profile of joint profiling dataset.

### *scMVP* accurately identified cell clusters from joint profiling cell line data

We next evaluated the extent to which the joint latent space inferred by scMVP reflected real biological similarity among cells. We benchmarked scMVP with single view scRNA-seq tools as Monocle3 [29], scVI [25], single view scATAC-seq tools as Monocle3 [29] and cisTopic [30], universal integration tools as MOFA+ [16], scAI [15], MultiVI [18], Cobolt [17], and paired dataset integration tools for multi-modalities in same cell as Seurat v4 WNN [19]. We assessed the accuracy of these methods by applying K-means clustering (using the same *k* as number of major cell types in dataset) and testing consistency with annotated cell labels.

**Fig. 3:**
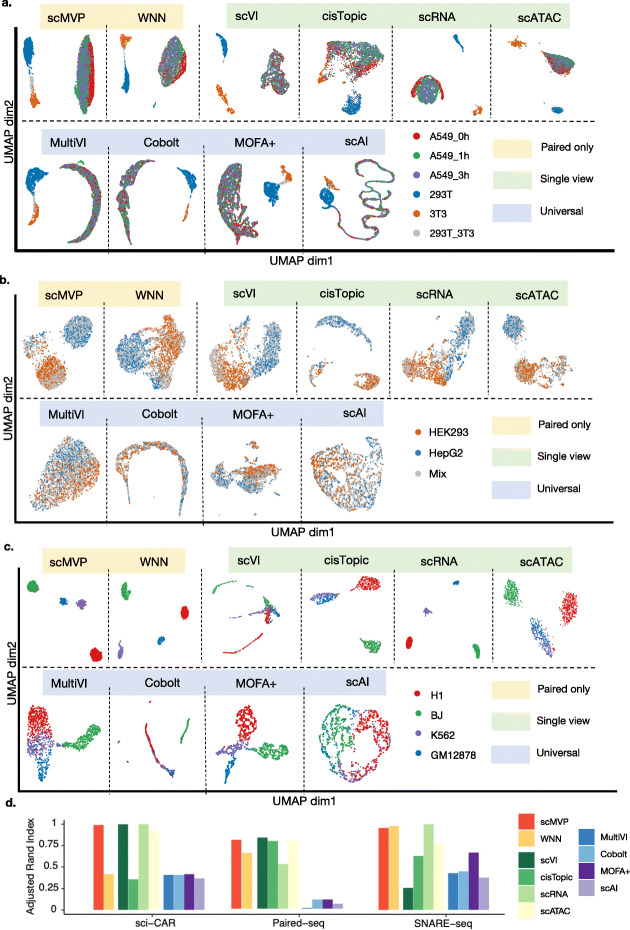
scMVP identifies accurate cell clusters from joint profiling cell line data. **a**–**c** Visualization of algorithms latent embeddings of three groups, algorithms specifically for joint modality datasets (shown as “Paired only”), algorithms of single omic (shown as “Single view”), and algorithms designed from both paired and unpaired datasets (shown as “Universal”) **a** UMAP visualization of scMVP, scVI, cisTopic, WNN, MultiVI, Cobolt, MOFA+, and scAI by Seurat v4 on the sci-CAR cell line dataset of A549, 293T, and 3T3 cells. **b** UMAP visualization of scMVP, scVI, cisTopic, WNN, MultiVI, Cobolt, MOFA+, and scAI by Seurat v4 on the Paired-seq cell line dataset of HEK293 and HepG2 cells. **c** UMAP visualization of scMVP, scVI, cisTopic, WNN, MultiVI, Cobolt, MOFA+, and scAI by Seurat v4 on the SNARE-seq cell line dataset of H1, BJ, K562, and GM12878 cells. **d** ARI scores for clustering on latent embeddings of benchmark algorithms

Firstly, we applied these algorithms to well-labeled cell line mixture data from sci-CAR, which included the 293T cell line, 3T3 cell line, 293T/3T3 cell mixture, and A549 cell line treated with dexamethasone (DEX) for 0 h, 1 h, and 3 h. scMVP, scVI, scRNA, and scATAC from Monocle3 grouped cells into three distinct clusters (293T, 3T3, and A549) from same cell annotations (Fig. 3a, Additional file 2: Table S1), and ARI scores of cells of annotated labels ranged from 0.92 to 1 (Additional file 1: Fig. S3, Additional file 1: Table S4), more accurate than cell clusters of WNN (0.42), cisTopic (0.36), and universal integration tools (0.37–0.42).

Next, we applied these algorithms to Paired-seq cell line data including two labelled cell types and their mixture. We first evaluated the cell clusters from these algorithms for cell annotated as HepG2 and HEK293. scMVP displayed a similar accuracy with scVI, cisTopic, and scATAC from Moncole3, better than Seurat v4 WNN and scRNA from Monocle3 but relatively lower than ARI scores of algorithms in sci-CAR dataset (Additional file 1: Fig. S3, Additional file 2: Table S4). However, all universal tools showed limit discrimination power of two cell types using their latent embedding with ARI scores ranged from 0.01 to 0.11, indicating the severe impact of data sparsity to current universal integration tools.

We further investigated UMAP visualization and found different number of cell subpopulations in these algorithms (Fig. 3b, Additional file 1: Table S2). Rather than the two cell clusters identified in UMAP results of other single-view algorithms and WNN, scMVP, and cisTopic yielded three cell clusters (Additional file 1: Fig. S2a-b), two of which identified as HEK293 cells and HepG2 cells, and another cluster that contained both cell types were largely consistent in two algorithms (Additional file 1: Fig. S2c). Then, we evaluated the gene and chromatin accessibility levels of each cell in the new cell cluster from scMVP and cisTopic. The new cluster showed relatively lower total RNA expression (*p* value<10^-10^) and relatively higher total expression in the scATAC-seq (*p* value<10^-10^) than the other two clusters (Additional file 1: Fig. 2d). These findings indicate that multi-omic integrated clustering in scMVP can be exploited to identify and cluster cells of abnormal state in either omic of joint profiling dataset after conventional methods that filter cells by extraordinarily high or low sequencing coverage threshold are used.

Then, we applied these algorithms to SNARE-seq cell line data including four labeled cell types. scMVP displayed a similar high accuracy with Seurat v4 WNN and scRNA from Monocle3 (Additional file 2: Table S3), which got four distinct subpopulations from same annotations in their UMAP visualization (Fig. 3c, Additional file 1: Fig. S2c). Rather than four clusters in scMVP and the other two algorithms, cisTopic, and scATAC from Monocle3 only got three clusters and grouped K562 and GM12878 into same cluster, which indicates that SNARE-seq could not distinguish K562 and GM12878 cells well with the single view of scATAC-seq, but could be well separated by integrated of both scRNA-seq and scATAC-seq by scMVP and Seurat v4 WNN. Four universal integration tools could not get four identical cell clusters in their latent embedding, although MOFA+ with better visualization discrimination of four cell types and higher clustering performance than other three integration tools.

We also evaluated the performance of algorithms designed for integration of different modalities in different cells as Seurat v3 [31] and Liger [32] for joint profiling cell line datasets. Seurat v3 cannot integrate scATAC and scRNA into consistent clusters in sci-CAR and Paired-seq cell line datasets and Liger cannot found consistent clusters in sci-CAR dataset (Additional file 1: Fig. S3-S5). And cells from same annotations cannot be distinguished into distinct subpopulations for Seurat v3 in SNARE-seq dataset and Liger in Paired-seq and SNARE-seq dataset, even if two algorithms can integrate the view of scRNA and scATAC from same cells.

Overall, analyzing joint profiling dataset with scMVP has proven to be helpful in identifying accurate grouping of cell clusters taking advantage of joint deep models and learning the characteristics from both layers of omic data.

### scMVP recovered major cell types in realistic datasets

**Fig. 4:**
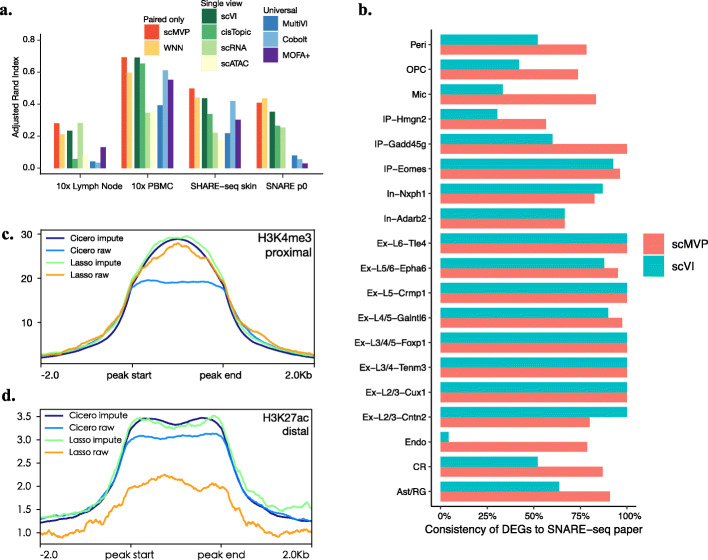
scMVP recovers major cell types in realistic datasets. **a** Adjusted rand index for nine benchmark algorithms in SNARE-seq mouse P0 dataset, 10X Genomics PBMC dataset, 10X Genomics Lymph Node dataset, and SHARE-seq mouse skin dataset. **b** Consistency between DEGs from SNARE-seq paper and top DEGs computed from scVI and scMVP scRNA imputation. **c** Aggregation profile of mouse forebrain P0 H3K4me3 ChIP-seq signal (ENCSR094TTT) in gene proximal cis-regulatory peaks computed by Cicero or LASSO for raw expression or scMVP imputed expression. **d** Aggregation profile of mouse forebrain P0 H3K27ac ChIP-seq signal (ENCFF695KNJ) in gene distal cis-regulatory peaks computed by cicero or LASSO for raw expression or scMVP imputed expression

To further examine the performance of scMVP on realistic joint profiling dataset, we used scMVP and other tools to analyze a 0-day postnatal (P0) mouse cerebral cortex dataset with 5081 cells generated by droplet-based SNARE-seq [8]. We first evaluated cells latent embedding and clustering accuracy of scMVP and other benchmark algorithms with reference cell annotations from Chen’s paper [8] (Fig. 4a, Additional file 1: Fig. S6, Additional file 2: Table S5). The ARI score of Monocle3 scATAC got only 0.002, and the UMAP visualization showed no discrimination among reference cell types, which indicates limited contribution of scATAC to cell clustering. However, both scMVP and WNN, which also integrated the data from scATAC, achieved higher clustering accuracy than other algorithms using only scRNA data of the joint profiling dataset. Among four universal integration tools, scAI could not complete the analysis within 48 h, and other three algorithms showed low clustering performance with ARI scores ranging from 0.03 to 0.08, suffering from low sequencing depth of the scATAC view of the dataset.

We next evaluated the performance of scMVP for 10X Multiome, which is the most popular multi-omics technology. We analyzed 7039 T cells in the 10X Lymph Node dataset with scMVP and other benchmark tools, as these T cells were well annotated by 10x Genomics, but difficult to distinguish the T cell subtypes by the view of scRNA or scATAC with ARI scores of 0.28 and 0.08 (Fig. 4a, Additional file 1: Fig. S7, Additional file 1: Table S5). The clustering accuracy of scMVP (0.28) was similar to the accuracy of Monocle3 scRNA, and higher than scVI (0.23), cisTopic (0.06), WNN (0.21), and universal integration tools, ranging from 0.03 to 0.13.

To test the performance scMVP on more complex realistic datasets, we then applied scMVP to two larger datasets; PBMC joint profiling dataset with 11,909 cells from 10X genomics multiome dataset, and mouse skin dataset with 34,773 cells from SHARE-seq dataset [11]. Compared to benchmark algorithms, scMVP showed consistent high agreement with the reference in both 10X PBMC dataset and SHARE-seq skin dataset (Fig. 4a, Additional file 1: Table S5), and most of the major references have corresponding cluster identified by scMVP (Additional file 1: Fig. S8-S9). Among four universal integration tools, scAI still could not complete the analysis within 48 h. However, MultiVI, Cobolt, and MOFA+ showed relative higher clustering performance compared to single view algorithms than their performance in SNARE P0 dataset and three cell line datasets, as the sequencing depth of 10X PBMC dataset and SHARE-seq skin dataset was much higher than SNARE P0 dataset and three cell line datasets.

Next, we evaluated scRNA and scATAC imputation from scMVP in downstream analysis of realistic dataset. We first performed differential gene analysis using gene imputations from scMVP and scVI with reference annotations. Compare to differential genes of each cell type in Chen’s paper [8], top differential genes computed by scMVP gene imputation were largely consistent in all reference cell types and were similar or higher than scVI gene imputation with the same threshold in most of the cell types (Fig. 4b).

We then performed cis-regulatory analysis for the mouse cerebral cortex P0 dataset. We used Cicero [13] to predict cis-regulatory interactions from scATAC-seq and also inferenced candidate CREs from scATAC-seq to differential genes in scRNA-seq using a LASSO method [9]. Cis-regulatory elements were predicted from raw count of scRNA-seq and scATAC-seq, and also the corresponding imputed expression from scMVP, and then evaluated with average signal enrichment of bulk forebrain P0 histone ChIP-seq in distinct peak set. Candidate regulatory peaks predicted from scMVP imputed expression with both Cicero and LASSO showed higher enrichment of H3K4me3 in translation start site (TSS) proximal regions than candidate peaks predicted from raw count (Fig. 4c). Also, H3K27ac and H3K4me1 signal showed higher enrichment in TSS distal peaks predicted from scMVP imputed expression than those distal peaks predicted from raw count (Fig. 4d, Additional file 1: Fig. S10). Thus, scMVP improved cis-regulatory elements prediction with scRNA-seq and scATAC-seq imputation in joint profiling dataset.

### scMVP facilitates trajectory inference with joint embedding latent features

Previous studies discovered the advantages of using latent embedding from deep generative models for scRNA-seq [25] or scATAC-seq [14], as well as using joint embedding of both scRNA-seq and scATAC-seq [19] to capture biological structure from single cell dataset. To further investigate the influence of joint embedding and deep generative model in scMVP to the latent embedding, we ran scMVP with scRNA or scATAC alone as input, which would not use joint embedding and reflect the performance of generative model in scMVP with scRNA-seq or scATAC-seq dataset, respectively. We also compared with latent embeddings from WNN, which would not use deep generative model and represent the performance of joint embeddings [19].

**Fig. 5:**
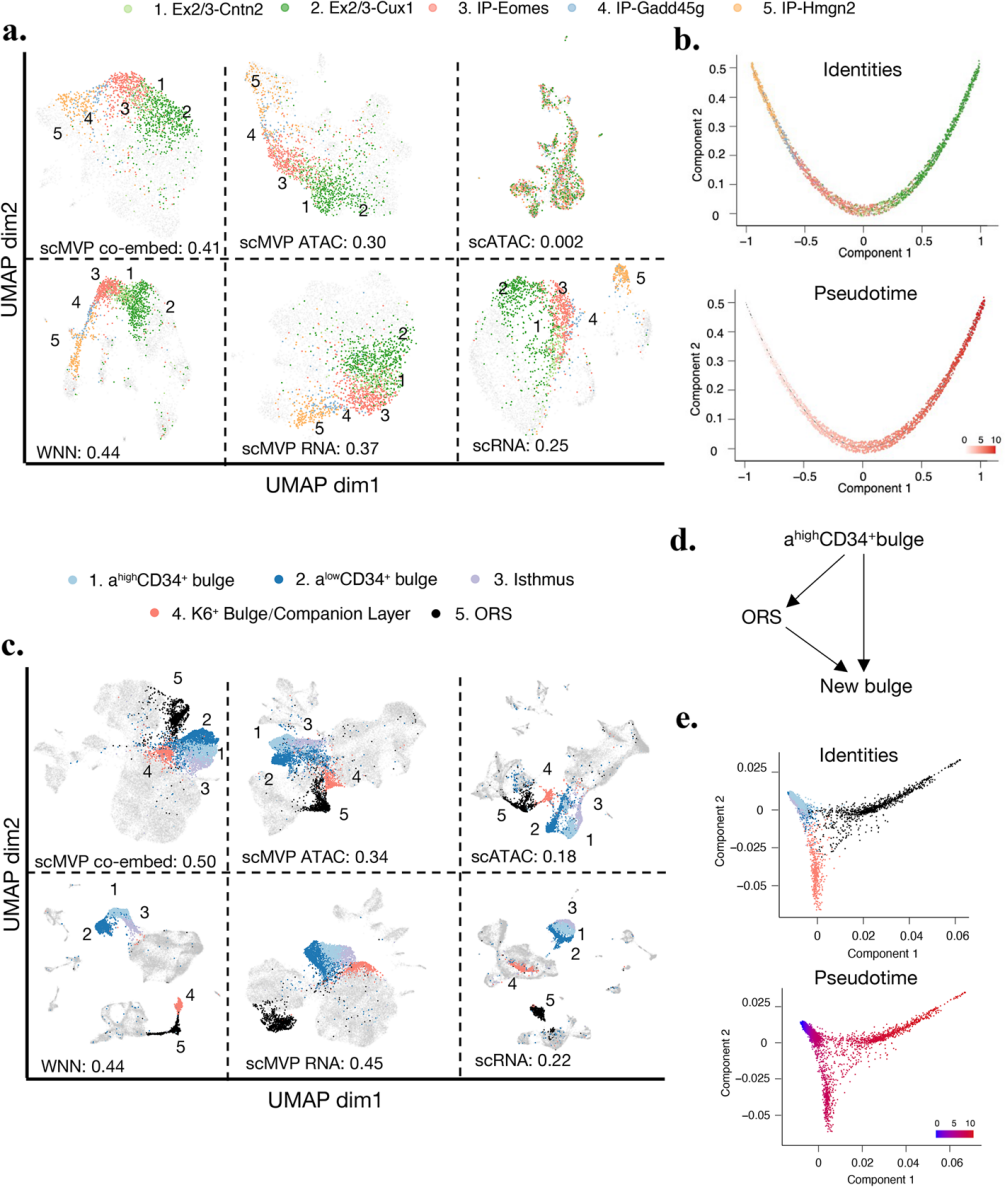
scMVP facilitates trajectory inference with joint embedding latent features. **a** UMAP visualization of SNARE-seq mouse cerebral cortex P0 dataset for 1469 cells (214 IP–Hmgn2, 99 IP–Gadd45g, 437 IP–Eomes, 177 Ex-L2/3–Cntn2, and 542 Ex-L2/3–Cux1) with scMVP with two omics, scRNA only, scATAC only, and Monocle3 with scRNA and scATAC. The ARI scores for clustering accuracy with each embedding were labelled in the subtitle of subplots. Ex excitatory neurons, IP intermediate progenitors. **b** Pseudotime trajectories constructed with scMVP joint embedding in Fig. 5a. Cells are colored according to pseudotime score (top) or cellular identity (bottom). **c** UMAP visualization of 4619 cells (1164 α^high^ CD34^+^ bulge, 1495 α^low^ CD34^+^ bulge, 537 Isthmus, 466 K6^+^ Bulge Companion Layer and 957 ORS) with scMVP with two omics, scRNA only, scATAC only, and Monocle3 with scRNA and scATAC. The ARI scores for clustering accuracy with each embedding were labelled in the subtitle of subplots. ORS outer root sheath. **d** Pseudotime diffusion map constructed with scMVP joint embedding in Fig. 5c. Cells are colored according to pseudotime score (top) or cellular identity (bottom). e The cell types shift during bulge cell development, referenced from SHARE-seq paper

We first evaluated the influence of joint embedding and deep generative model on SNARE P0 dataset. Compared to scRNA (ARI=0.25) and scATAC (ARI=0.002) raw data using Monocle3, scMVP using scRNA or scATAC as input would improve the clustering accuracy to 0.37 and 0.30, which was consistent with previous report for deep generative models [14, 25] but still lower than performance of scMVP (ARI=0.41) and WNN (ARI=0.44) using joint embedding (Fig. 5a). We then focused on the transition of intermediate progenitors (IP) to upper-layer excitatory neurons (Ex). The relative position of five cell types in UMAP visualizations was consistent with development order [8] in all latent embeddings except for scATAC raw data, which could hardly distinguish any cell type in the latent embedding (Fig. 5a). Using diffusion map [33] , we next ordered these cells along a pseudotime trajectory with joint embedding. The development order of five cell types was largely consistent to the order in pseudotime (Fig. 5b). We found Sox6 which encodes a transcript factor for maintenance of neuron precursor cells [34], and membrane-protein-encoding Mlc1 showed a decline of gene expression along the trajectory of neuron differentiation (Additional file 1: Fig. S11a-b). And the gene Khdrbs2, which encodes an RNA-binding protein involved in alternative splicing, along with its target gene Nrxn1 [35] showed a similar rise along same trajectory (Additional file 1: Fig. S11c-d). The alterations of gene and promoter expression along joint embedding trajectory were similar with same cellular trajectory in SNARE-seq paper [8].

Next, we performed same analysis on SHARE-seq skin dataset. scMVP with single-channel input would improve the clustering accuracy of raw data from 0.22 to 0.45 for scRNA and 0.18 to 0.34 for scATAC, revealing consistent advantage of using deep generative model to capture biological cell types in the latent embedding. Clustering accuracy of scMVP with scRNA input was similar to WNN (ARI=0.44), but still lower than joint embedding from scMVP (ARI=0.50) (Fig. 5c). We then focused on development of bulge stem cells to new bulge cells. Consistency with difference of UMAP latent embedding between scRNA and scATAC in SHARE-seq paper [11], the two CD34^+^ bulge cells and Isthmus cells were adjacent to K6^+^ Bulge/Companion Layer in UMAP visualization of scATAC raw data latent embedding, but separated in the latent embedding of scRNA raw data (Fig. 5c). And the ORS (outer root sheath) cells were partially linked with K6^+^ Bulge/Companion Layer in UMAP visualization of scATAC raw data, consistent with order of cell type shifts in bulge development [11] (Fig. 5d). The relative position of cell types was retained in both scMVP with scATAC input and scMVP joint embedding, indicating the reserved biological cell type structure during deep generation model. The WNN captured the relative connection between ORS and K6^+^ Bulge/Companion Layer, which was missed in latent embedding of scRNA from raw data and scMVP, but failed to capture cell type shifts from α^high^ CD34^+^ bulge, α^low^ CD34^+^ bulge, and Isthmus in the scATAC latent embedding with simply joint embedding. We then perform trajectory analysis on scMVP joint embedding of developmental bulge cells. We found the diffusion map and pseudotime detecting both two paths from α^high^ CD34^+^ bulge to new bulge cells (Fig. 5e). Overall, deep generation model along with joint embedding in scMVP could effectively improve clustering accuracy and capture the biological structure hidden in scRNA or scATAC of the joint profiling dataset.

## Discussion

scMVP was designed as a ready-to-use deep generative model to handle sequencing data that simultaneously measure gene expression and chromatin accessibility in the same cell, including SNARE-seq [8], sci-CAR [9], Paired-seq [10], SHARE-seq [11], and 10X multiome. Two major challenges in the analysis of scRNA and scATAC joint profiling data are addressed by scMVP. The first challenge is how to overcome difficulties in processing a very sparse and high dimensional data matrix, as the sequencing data throughput of the latest joint profiling methods is much lower than the throughput of single-modality scRNA-seq or scATAC-seq data. Recently, several algorithms [15–18] were developed to analyze both joint modality dataset as 10X multiome PBMC dataset and unpaired datasets and showed relative high performance on high quality joint profiling dataset. However, the performance of these universal integration algorithms showed limited explanation power in their latent embedding when applied to more sparse and noisy joint profiling datasets, which may impede their application if the joint profiling dataset is not as “good” as 10X multiome PBMC dataset. To provide a generally application to joint profiling datasets from different technique platform, scMVP utilized the multi-head self-attention-based transformer structures in the ATAC module and cycle-GAN like module to enhance the signal from both view of joint modality dataset. The output layer of scMVP with appropriate distribution can impute genes and peaks from the common latent embedding layer by maximizing the likelihood of the bimodal omic data. The scRNA-seq imputation of scMVP in cell line datasets showed higher consistency to gene expression of bulk cell line RNA-seq than raw count and similar or better consistency with scVI imputation [25], revealing the advantage of generative models in gene imputation for scRNA-seq of joint profiling data (Fig. 2a). Also, the scATAC-seq imputation of scMVP identified more ATAC-seq or DNase-seq peaks in corresponding cell line than raw count of scATAC-seq with similar accuracy (Fig. 2b). Additionally, CREs predicted from scRNA-seq and scATAC-seq imputation displayed higher regulatory potential than CREs predicted from raw count of scRNA-seq and scATAC-seq in the SNARE-seq cerebral cortex dataset (Fig. 4c–d), indicating the availability of joint imputation of scMVP for joint profiling datasets. The second challenge is how to utilize two omic datasets for single-cell data analyses, such as cell denoising, cell clustering, and development trajectory inference rather than conventional independent analysis of scRNA-seq and scATAC-seq followed by integration or anchoring of the two omic datasets between similar cell clusters, as common integration tools as Seurat v3 and Liger were not applicable for integration of two omic datasets in the same cell (Additional file 1: Fig. S3-5). Taking advantages of multi-modal deep models, scMVP can directly perform these analyses on common latent code in an embedding layer and provides accurate cell clusters in all cell line datasets and realistic datasets, which is more robust than other single cell analysis tools (Figs. 3 and 4a, b).

Different from other single-cell deep generation algorithms, scMVP utilized a joint embedding structure. We then investigated the influence of joint embedding and deep generative model in scMVP to capture biological cell type structure. For both SNARE-seq mouse cerebral cortex P0 dataset and SHARE-seq mouse skin dataset, the characteristic of deep generation model for both scRNA and scATAC will improve the clustering accuracy and retain the relative cell type structure in raw data. And the joint embedding of WNN from two omics would also improve cell clustering and retain the biological structure in scRNA data when the scATAC data of SNARE-seq P0 showed limited contribution to latent embedding (Fig. 5c). However, when the biological structure of scRNA and scATAC latent embeddings differs in SHARE-seq skin dataset, we found deep generative model for scRNA or scATAC would also learn the biological structure from latent embeddings in respective raw data (Fig. 5d). Joint embedding of WNN would also improve the clustering accuracy, but it could not capture the expected cell type order from scATAC data (Fig. 5d, e). Benefit from continuous sampling attributes in deep generative architecture and integration attributes in multi-channel architecture, scMVP not only improved the cell clustering, but also learned the biological structure from the scATAC, and inferred cell trajectory from both omics data.

Finally, it is worth noting that the multi-modal deep generation models described here could also be extended to parallel profiling of other epigenomic data, such as DNA methylation level [36, 37], TFs [38], and spatial chromatin structure [31]. Overall, scMVP was designed as a general, flexible, and extensible framework to reconcile heterogeneity across multiple omic datasets while remaining robust to the substantial amount of missing data inherent in joint RNA and ATAC single-cell sequencing experiments. The multi-channel encoder architecture of scMVP could also be transformed for use in traditional single-cell multi-omic data analyses [39].

## Conclusions

In this study, we introduced scMVP, a non-symmetric deep generative model designed for comprehensive handling sequencing datasets that simultaneously measure gene expression and chromatin accessibility in the same cell. We applied scMVP to datasets from various joint profiling techniques and found scMVP as robust and effective tool in downstream analysis tasks with both joint latent embedding and separate imputations from two omics.

## Methods

### The generative model of scMVP

For joint profiles of scRNA-seq and scATAC-seq data, the expression profiles of RNA and TF-IDF transformed ATAC [40], which convert original binary peaks into continuous value by weighting each peak with its occurring frequency, are represented as gene expression vector ***x***_*i*_ ∈ *R*^∣*G*∣^ and ATAC peak vector ***y***_*i*_ ∈ *R*^∣*P*∣^,*i* = 1, 2, …, *N*, where *G* is the number of all detected genes, *P* is the corresponding number of detected peaks, and *N* is the total number of cells.

Attempting to capture the biological physiology of the cells of interest (e.g., cell types, developmental state), a multi-view generative model is built to recover the scRNA profile ***x***_*i*_ and scATAC profile ***y***_*i*_ from a common latent embedding ***z***_***i***_ ∈ *R*^*D*^ (the dimension *D* ≪ min(|*G*|, ∣ *P*∣), where the latent code ***z***_***i***_ follows a GMM-based prior distribution and ***x***_*i*_ and ***y***_*i*_ each follow a negative binomial (NB) distribution and zero-inflated Poisson distribution [41]. The Poisson distribution is better to fit the signal counts of TF-IDF transformed scATAC chromatin accessibility [40] rather than the regular binary transformation. Due to the extreme sparsity of scATAC dataset, we use zero-inflated Poisson for scATAC peaks in current joint sequencing technique. That is:

1 $$ \mathrm{p}\left(\mathrm{c}\right)= Cat\left(\boldsymbol{\pi} \right)=\prod \limits_{k=1}^K{\pi_k}^{c_k},\boldsymbol{\pi} =\left[{\pi}_1,{\pi}_2,\dots, {\pi}_K\right] $$

2 $$ \mathrm{p}\left(\mathrm{z}|\mathrm{c}\right)=N\left(z|{\mu}_c,{\sigma}_c\mathrm{I}\right)=\frac{1}{\sqrt{2\pi }{\sigma}_c}\ {e}^{\left(-\frac{{\left(z-{\mu}_c\right)}^2}{2{\sigma}_c^2}\right)\kern0.5em } $$

3 $$ {\alpha}_x,{\beta}_x={Decoder}_x(z) $$

4 $$ p\left({\mu}_x|{\alpha}_x,{\beta}_x\right)= Gamma\left({\alpha}_x,{\beta}_x\right)=\frac{{\beta_x}^{\alpha_x}{\overline{x}}^{\alpha_x-1}{e}^{-{\beta}_x\overline{x}}}{\Gamma \left({\alpha}_x\right)} $$

5 $$ p\left(\mathrm{x}|{\mu}_x\right)= Poisson\left({\mu}_x\right)=\frac{{\mu_x}^{\mathrm{x}}}{\mathrm{x}!}{e}^{-{\mu}_x} $$

6 $$ {\mu}_y,{\tau}_y={Decoder}_y(z) $$

7 $$ p\left(\overline{y}|{\mu}_y\right)= Poisson\left({\mu}_y\right)=\frac{{\mu_y}^{\overline{y}}}{\overline{y}!}{e}^{-{\mu}_y} $$

8 $$ \mathrm{p}\left({\omega}_y|{\tau}_y\right)= Bernoulli\left({\tau}_y\right)={\tau_y}^{\omega_y}{\left(1-{\tau}_y\right)}^{1-{\omega}_y} $$

9 $$ \mathrm{p}\left(\mathrm{y}|\overline{y},{\omega}_y\right)={\left[\ p\left(\overline{y}|{\mu}_y\right)\ast \mathrm{p}\left({\omega}_y=1|{\tau}_y\right)\right]}_{y>0}+{\left[\mathrm{p}\left({\omega}_y=0|{\tau}_y\right)+p\left(\overline{y}|{\mu}_y\right)\ast \mathrm{p}\left({\omega}_y=1|{\tau}_y\right)\right]}_{y=0} $$

Here, *c* represents one of the *K* components(clusters) of Gaussian mixture distribution, which is extract from a categorical distribution with probability *π*_*c*_, then the common embedding latent variable *z* is derived from the component *c* with a probability p(z| c) = N(*z*| *μ*_*c*_, *σ*_*c*_I), which means the latent variable *z* associated cells is belonged into a specific cluster (cell type) *c*. Then, a two-channels decode network is used to generate the parameters of the NB and ZIP distribution to reconstruct the original observed *x* (RNA) and TF-IDF transformed *y* (ATAC) from the common latent variable *z*. In this paper, we decompose the NB distribution as a composite of a Gamma distribution with shape parameter *α*_*x*_ and scale parameter *β*_*x*_, a Poisson distribution with mean parameter *μ*_*x*_ given by the Gamma distribution sampling, in which the gamma distribution captures the real distribution of expression values, the Poisson distribution simulates the sequencing bias. As a result, the RNA counts can be imputed with the mean of the Poisson distribution. Similarly, the ZIP distribution is decomposed as a Poisson distribution and a Bernoulli distribution, and the mean *μ*_*y*_ of Poisson distribution is worked here as the imputation of scATAC-seq data. To coordinate the potential correlations between the RNA and ATAC data in the same cell, we introduce an attention module to weight the two decoder channels using the probabilities of latent variable *z* belonging to each of the *K* cluster components (Fig. 1a and Additional file 1: Fig. S12). In addition, to make sure the embedding and clustering consistency between the original and imputed data, we design a cycle consistency module to match each layer of latent variables from the imputed RNA and ATAC data, respectively, with the joint embedding latent variable from the original data (Fig. 1a and Additional file 1: Fig. S12, S14).

Specifically, the *Decoder*_*y*_(*z*) is designed as a self-attention-based transformer subnetwork to capture weak and genome-wide correlation from sparse and high-dimensional (>10^5^) scATAC data [21], that is:

10 $$ {Decoder}_y(z)= LayerNorm\left( BatchNorm\left( MLP(z)\right)+\mathrm{MultiHead}\left(\mathrm{Q}\left( BatchNorm\left( MLP(z)\right)\right),\mathrm{K}\left( BatchNorm\left( MLP(z)\right)\right),\mathrm{V}\left( BatchNorm\left( MLP(z)\right)\right)\right)\right)\ast \kern0.5em \mathrm{Softmax}\left( MLP\left(p\left(z|c\right)\right)\right) $$

11 $$ \mathrm{MultiHead}\left(\mathrm{Q},\mathrm{K},\mathrm{V}\right)=\mathrm{Concat}\left({head}_1,\dots, {head}_h\right){W}^O $$

12 $$ {head}_i= Attention\left(Q{W}_i^Q,K{W}_i^K,V{W}_i^V\right)= softmax\left(\frac{\left(Q{W}_i^Q\right){\left(K{W}_i^K\right)}^T}{\sqrt{d_k}}\right)V{W}_i^V $$

As shown in formula 10, the *Decoder*_*y*_(*z*) is cascaded by a multilayer perceptron (MLP), a batch normalization, a multi-head self-attention-guided skip connection module (similar with the Resnet block) and a layer normalization, which is then weighted by Softmax(*MLP*(*p*(*z*| *c*))), functioned as the additional cell cluster-indicated attention module to recover the cell-type specific semantic information (Additional file 1: Fig. S12). In detail, we firstly produce queries (Q), keys (K) and values (V) matrixes for self-attention module using *BatchNorm*(*MLP*(*z*)), the output of batch-normalized multilayer perceptron, and then split each of three matrixes into the *i*th of *h* heads by multiplying each head specific transformation weight matrix: ${W}_i^Q$,${W}_i^K$, and ${W}_i^V$, respectively. Next, the *i*th head-indicated values $V{W}_i^V$ is weighted by the $softmax\left(\frac{\left(Q{W}_i^Q\right){\left(K{W}_i^K\right)}^T}{\sqrt{d_k}}\right)$, the activated correlation attention matrix between the *i*th queries and keys, where the *d*_*k*_ is the scale factor [21] (formula 12). Finally, all the *h* heads are concatenated together to decode the latent embedding *z* as the ZIP distribution parameters *μ*_*y*_, *τ*_*y*_ for generating ATAC profile (formula 6) with a transformation matrix *W*^*O*^ and a skip-connection layer (formula 11). Contrarily, the RNA generating/decoder subnetwork *Decoder*_*x*_(*z*) utilizes a canonical mask attention structure by cascading a multilayer perceptron, a layer normalization, a batch normalization, and an attention module (Additional file 1: Fig. S12), which can be presented as follows:

13 $$ {Decoder}_x(z)= MLP\left( BatchNorm\left( LayerNorm\left( MLP(z)\right)\right)\ast \mathrm{Softmax}\left( MLP(z)\right)\right)\ast \kern16.5em \mathrm{Softmax}\left( MLP\left(p\left(z|c\right)\right)\right) $$

This branch is also weighted by Softmax(*MLP*(*p*(*z*| *c*))), the additional cell cluster-indicated attention module.

scMVP model is optimized by maximizing the log likelihood probability of the generated scRNA and ATAC data according to variational Bayesian inference [42]:

14 $$ {\displaystyle \begin{array}{c}\log p\left(x,y\right)=\log \int {\sum}_cp\left(x,y,z,c\right) dz\\ {}\ge \int {\sum}_cq\left(z,c|x,y\right)\ast \log \frac{p\left(x,y,z,c\right)}{q\left(z,c|x,y\right)}\\ {}={E}_{q\left(z,c|x,y\right)}\left[\log \frac{p\left(x,y,z,c\right)}{q\left(z,c|x,y\right)}\right]={\mathcal{L}}_{elbo}\left(x,y\right)\end{array}} $$

where *q*(*z*, *c*| *x*, *y*) is the introduction variational distribution. According to our network structure and the mean field theory [42], we can get:

15 $$ p\left(x,y,z,c\right)=p\left(x|z,c\right)p\left(y|z,c\right)p\left(c|z\right)p(z) $$

16 $$ q\left(z,c|x,y\right)=q\left(z|x,y\right)q\left(c|z\right) $$

Here, *p*(*x*| *z*, *c*) is worked as a NB distribution and generated by *p*(x| *μ*_*x*_) ∗ *p*(*μ*_*x*_| *α*_*x*_, *β*_*x*_), while *p*(*y*| *z*, *c*) is worked as a ZIP distribution and generated by $\mathrm{p}\left(\mathrm{y}|\overline{y},{\omega}_y\right)\ast \mathrm{p}\left({\omega}_y|{\tau}_y\right)\ast p\left(\overline{y}|{\mu}_y\right)$ (see formula 1–9), and all distribution parameters as *α*_*x*_, *β*_*x*_, *τ*_*y*_, and *μ*_*y*_ are generated from decoder network. The *q*(*z*| *x*, *y*) is inferenced from the joint encoder network in scMVP model, which is composed of a mask attention-based scRNA embedding subnetwork and transformer self-attention-based scATAC embedding subnetwork, as each of them has a similar structure with the corresponding decoder subnetwork (Additional file 1: Fig. S13a).

Then, the variational lower bound can be represented as follows:

17 $$ \kern2.5em {\mathcal{L}}_{elbo}\left(x,y\right)={E}_{q\left(z|x,y\right)q\left(c|z\right)}\left[p\left(x|z,c\right)\right]+{E}_{q\left(z|x,y\right)q\left(c|z\right)}\left[p\left(y|z,c\right)\right]-{D}_{KL}\left(p(z)\Big\Vert q\left(z|x,y\right)\right)-{D}_{KL}\left(p\left(c|z\right)\Big\Vert q\left(c|z\right)\right) $$

To further improve the performance of scMVP model to extreme sparse dataset as joint profiling dataset, we introduce a cycle-GAN like clustering consistency auxiliary network to coordinate the latent embedding of each scMVP imputed profile with the joint embedding from raw profile. Due to the different characteristics of scRNA data and scATAC data, we applied transformer self-attention-based imputed embedding for scATAC cycling workflow and mask attention-based module for scRNA cycling workflow (Additional file 1: Fig. S13 b, c and S14). Similar with the cycle consistency loss used in cycle-GAN model, the clustering consistency loss can be represented as the Kullback-Leibeler divergence of the embedding between the imputed and original data:

18 $$ \kern2em {\mathcal{L}}_{consistency}\left(x,y,{x}_{impute},{y}_{impute}\right)={D}_{KL}\left(q\left(z|x,y\right)\parallel q\left(z|{x}_{impute}\right)\right)+{D}_{KL}\Big(q\left(z|x,y\right)\parallel q\left(z|{y}_{impute}\right) $$

where *q*(*z*| *x*_*impute*_) and *q*(*z*| *y*_*impute*_) represent the latent embedding of imputed scRNA and scATAC, respectively.

To maximize ${\mathcal{L}}_{elbo}\left(x,y\right)$, the independent component *D*_*KL*_(*p*(*c*| *z*)‖*q*(*c*| *z*)) ≡ 0 should be satisfied (in fact, the discrete variable *c* is depended only on *z*); and considering the clustering consistency loss ${\mathcal{L}}_{consistency}\left(x,y,{x}_{impute},{y}_{impute}\right)$, we use a constrained optimization process to solve ${\mathcal{L}}_{elbo}\left(x,y\right)$:

19 $$ {\mathcal{L}}_{elbo}^{\prime }={E}_{q\left(z|x,y\right)q\left(c|z\right)}\left[p\left(x|z,c\right)\right]+{E}_{q\left(z|x,y\right)q\left(c|z\right)}\left[p\left(y|z,c\right)\right]-{D}_{KL}\left(p(z)\ \Big\Vert\ q\left(z|x,y\right)\right)-{D}_{KL}\left(q\left(z|x,y\right)\parallel q\left(z|{x}_{impute}\right)\right)-{D}_{KL}\left(q\left(z|x,y\right)\parallel q\left(z|{y}_{impute}\right)\right) $$

20 $$ s.t.\kern0.5em p\left(c|z\right)=q\left(c|z\right)=\frac{p\left(z|c\right)p(c)}{\sum_{c^{\prime }=1}^Kp\left({z}^{\prime }|{c}^{\prime}\right)p\left({c}^{\prime}\right)} $$

In practice, the parameters of variational distribution *q*(*z*| *x*, *y*) is implemented in a two-channel encoder network concatenated with a joint embedding layer, the distribution parameters of *p*(*x*| *z*, *c*) and *p*(*y*| *z*, *c*) are generated through the decoder network as shown in formulas (1–9). Then, *E*_*q*(*z*| *x*, *y*)_[*p*(*x*| *z*, *c*)] and *E*_*q*(*z*| *x*, *y*)_[*p*(*x*| *z*, *c*)] represent the log likelihood of reconstructed scRNA-seq and scATAC-seq data, respectively, and the Kullback-Leibeler divergence *D*_*KL*_(*p*(*z*)‖*q*(*z*| *x*, *y*)) regularizes the latent variable *z* into one of the *K* Gaussian distributions for cell type identification, and the parameters of *p*(*z*| *c*) and *p*(*c*) are estimated by the gradient back-propagation of decoder network.

In our study, scMVP consists of a two-channel encoder network and a two-channel decoder to integrate the information from scRNA-seq and scATAC-seq, and the input dimension of each channel is determined by the gene and peak number. Different from the network layer design in scVI [25] and SCALE [14], we used a mask attention channel for RNA branch and a self-attention channel for ATAC branch to identify the cell type associated information and capture the intra-omics distal correlation. Specifically, RNA branch of encoder sequentially concatenates 128-dimensional hidden layer, a layer normalization layer, a batch normalization layer, and an output Relu activation layer, which is weighted by a mask attention tensor generated from the first 128-dimensional hidden layer. The ATAC branch of encoder sequentially concatenates a 128-dimensional hidden layer, a batch normalization layer, a Relu activation layer, and a multi-heads self-attention layer, which is designed as 8 self-attention heads and each head takes 16-dimension feature in this study, and a layer normalization. The output two channels are combined together to form a shared linear layer (256 dimensions). Finally, two cascaded 128-dimension linear layers are used to produce the mean and variance of a normal distribution *N*(z| μ, σ) for the 10-dimensional common latent variable *z* (Additional file 1: Fig. S13). After a reparameterization trick with *z* = *μ* + σ*N*(0, 1), which is a specific sampling scheme from the variational distribution, and used to approximate the expectation of *q*(*z*| *x*, *y*), a two-channel decoder is employed to determine the distribution parameters of NB and ZIP for the reconstruction of scRNA-seq and scATAC-seq, which utilize a similar network structure with the encoder network except an attention module. The attention module receives the *p*(*c*| *z*) for all *K* components as input, by a linear layer (128 dimensions), and then weights the last layer of each decoder channel with a SoftMax activation function (Additional file 1: Fig. S12). Finally, the imputed scRNA-seq and scATAC-seq data are fed back two single-channel encoders to produce the imputed embedding for clustering consistency evaluation, and those two encoders have the same structure with each omic-specific encoder branch and are trained with the joint encoder simultaneously. The raw scRNA-seq and scATAC-seq data are also used to train for both subnetworks, avoiding the possible overfitting from cycling clustering consistency training of the imputed data.

In addition, the cluster number *K* should be user predefined or specified by the rank of cell-cell correlation matrix. The GMM algorithm is used to estimate the initial parameters of the Gaussian mixture prior distribution [43]. Our model is trained using the Adam optimizer with a mini-batch of 128, learning rate 5.0e−3, and the maximum number of iterations is 30. The neural network of scMVP is implemented with PyTorch, and the GMM is constructed with the scikit-learn package [44] from python.

### Data analysis and model evaluation

#### Cell line pre-processing and visualization

The sci-CAR cell line dataset was derived from 293T, 3T3, 293T/3T3 cell mixtures, and A549 cell lines treated with DEX for 0 h, 1 h, and 3 h [9]. For the sci-CAR dataset, only co-assay cells were used for further analysis, and cells with fewer than 200 peaks or genes and peaks or genes with fewer than 10 cells were removed from further analysis. The Paired-seq cell line dataset was derived from HEK293, HepG2, and their cell line mixture [10]. For Paired-seq dataset, cells with fewer than 200 peaks or genes, peaks, or genes with fewer than 10 cells or peaks with more than 336 cells were removed from further analysis. The downsampled sciCAR and Paired-seq cell line datasets were used for training epochs evaluation. The SNARE-seq cell line dataset was derived from H1, BJ, K562, and GM12878 [8]. For SNARE-seq dataset, cells with fewer than 200 peaks or genes and peaks or genes with fewer than 10 cells were removed from further analysis. For model performance evaluation, we used replicate 3 of GM12878 cell line with 67,418 cells from SHARE-seq dataset [11]. Cells were filtered with same threshold and top 8000 genes/23,000 peaks were used for memory and training time benchmark. We sampled 1000, 2000, 5000, 10,000, 20,000, 50,000, and 100,000 cells from SHARE-seq GM12878 dataset in a put-back way. For batch removal evaluation, we used 2973 cells and 8803 cells from replicate 2 and replicate3 of GM12878 cell line from SHARE-seq dataset. We used Seurat [19] for data pre-processing of all datasets. UMAP visualization and clustering of the scATAC profiles was performed using Monocle3 [29] and cisTopic [30], and scRNA profiles of the cell lines were produced with the same analysis using Monocle 3 [29] and scVI [25]. UMAP visualization of multi-view integrations were also processed with Seurat v3 [31], Liger [32], Seurat v4 WNN [19], MOFA+ [16], scAI [15], MultiVI [18], and Cobolt [17].

#### Cell line clustering and imputation evaluation

We used the metric of adjusted Rand Index (ARI) for clustering comparison of algorithms as described in previous literature [14, 25]. Cells derived from unique cell line were used for clustering benchmark in sci-CAR dataset of 3 cell types, Paired-seq of 2 cell types, and SNARE-seq of 4 cell types.

We used gene quantifications of bulk cell line RNA-seq for gene imputation evaluation. Gene count files of A549 cell lines treated with DEX for 0h (ENCSR632DQP), 1h (ENCSR656FIH), 3h (ENCSR624RID) used in sci-CAR dataset, HepG2 cell line (ENCSR058OSL) used in Paired-seq dataset and H1 (ENCSR670WQY), BJ (ENCSR000COP), K562 (ENCSR530NHO), and GM12878 (ENCSR000CPO) cell lines used in SNARE-seq dataset were downloaded from ENCODE3 data portal [45] for gene imputation benchmark. We also downloaded DNase-seq signal files for H1 (ENCSR000EMU) and BJ (ENCSR000EME), and ATAC-seq signal files for K562 (ENCSR868FGK) and GM12878 (ENCSR095QNB) ENCODE3 data portal. Bulk DNase-seq signal and bulk ATAC-seq signal in single-cell ATAC-seq peaks were computed with UCSC tools bigwigAverageOverBed [46], and single-cell ATAC-seq peaks with signal over certain threshold were used as valid peaks in bulk DNase-seq and ATAC-seq. One-tailed *t* test was used to estimate the significance of true peak count and true peak ratio of scMVP scATAC imputation higher than raw scATAC counts.

#### Realistic datasets pre-processing, clustering evaluation, and trajectory inference

The same pre-processing procedures and same algorithms were used for three realistic datasets with cell line dataset. For clustering evaluation, reference cell annotations of 10X Genomics PBMC dataset and fresh frozen lymph node with B cell lymphoma dataset (10x Lymph Node dataset) were downloaded from 10X Genomics website (www.10xgenomics.com/resources/datasets), as annotation of SHARE-seq skin dataset was downloaded from SHARE-seq paper [11]. Reference cell annotations of SNARE-seq mouse cerebral cortex P0 dataset was provided by Prof. Kun Zhang [8]. Differential gene analysis of SNARE-seq P0 dataset were performed by scanpy [47] using both scMVP and scVI scRNA imputation and reference cell annotations. Consistency of DEGs in each cell type was calculated by number of top DEGs from scMVP or scVI overlap with distinct SNARE paper DEGs divided by number of SNARE paper DEGs used for gene imputation. Cellular trajectory and pseudotime were computed with joint latent embeddings of SNARE-seq and SHARE-seq dataset by function DiffusionMap in R package destiny [48].

#### Evaluation of CRE prediction in the SNARE-seq P0 dataset

Candidate cis-regulatory elements were predicted from original and scMVP imputed scATAC data using Cicero [13] with default parameter. For each gene, we also computed correlations between its expression and the binary accessibility of all peaks located 100 kilobases (kb) of its transcriptional start site (TSS) using LASSO (least absolute shrinkage and selection operator) with R package glmnet [49]. Gene proximal peaks for each peak list were defined as peaks located within 2 kb upstream to 500kb downstream of transcription start sites (TSS), as other peaks were defined as gene distal peaks. H3K27ac (ENCSR094TTT), H3K4me1 (ENCSR465PLB), and H3K4me3 (ENCSR258YWW) ChIP-seq of mouse forebrain P0 files were downloaded from the ENCODE3 data portal [45]. Aggregation of the H3K27ac signal, H3K4me1 signal, and H3K4me3 signal on both gene proximal and distal CREs was performed with deepTools2 [50].

##### Peer review information

Barbara Cheifet was the primary editor of this article and managed its editorial process and peer review in collaboration with the rest of the editorial team.

##### Review history

This manuscript was previously reviewed at another journal, no review history is available.

## Supplementary Information

**Additional file 1: Supplementary Figures S1-S14**.

**Additional file 2: Supplementary Tables S1-S10**.

## Data Availability

scMVP is implemented as Python packages, and it is freely available under the MIT license on GitHub (https://github.com/bm2-lab/scMVP) [51]. The specific scMVP release used for the results presented in this manuscript is archived on zenodo [52]. The repository includes vignettes, source code, pre-processed datasets and pre-trained models to reproduce the analyses presented in this article. The datasets analyzed in this study are available from the Gene Expression Omnibus (GEO) repository under the following accession numbers: GSE126074 [8], GSE130399 [10], GSE140203 [11], GSM3271040 [9], and GSM3271041 [9].
