# Supplementary material for: A deep generative model for multi-view profiling of single-cell RNA-seq and ATAC-seq data
Source: Genome Biol. 2022 Jan 12;23:20. doi: 10.1186/s13059-021-02595-6 (PMC8756637; doi:10.1186/s13059-021-02595-6)
Supplement: Supplementary file 1 — Additional file 1: Supplementary Figures S1-S14. [file 13059_2021_2595_MOESM1_ESM.pdf]

A deep generative model for multi-view profiling of single-cell RNA-seq and ATAC-seq data

Additional file 1: Supplementary figures

Gaoyang Li[#], Shaliu Fu[#], Chenyu Zhu, Bin Duan, Chen Tang, Xiaohan Chen, Guohui Chuai, Ping Wang*, Qi Liu*

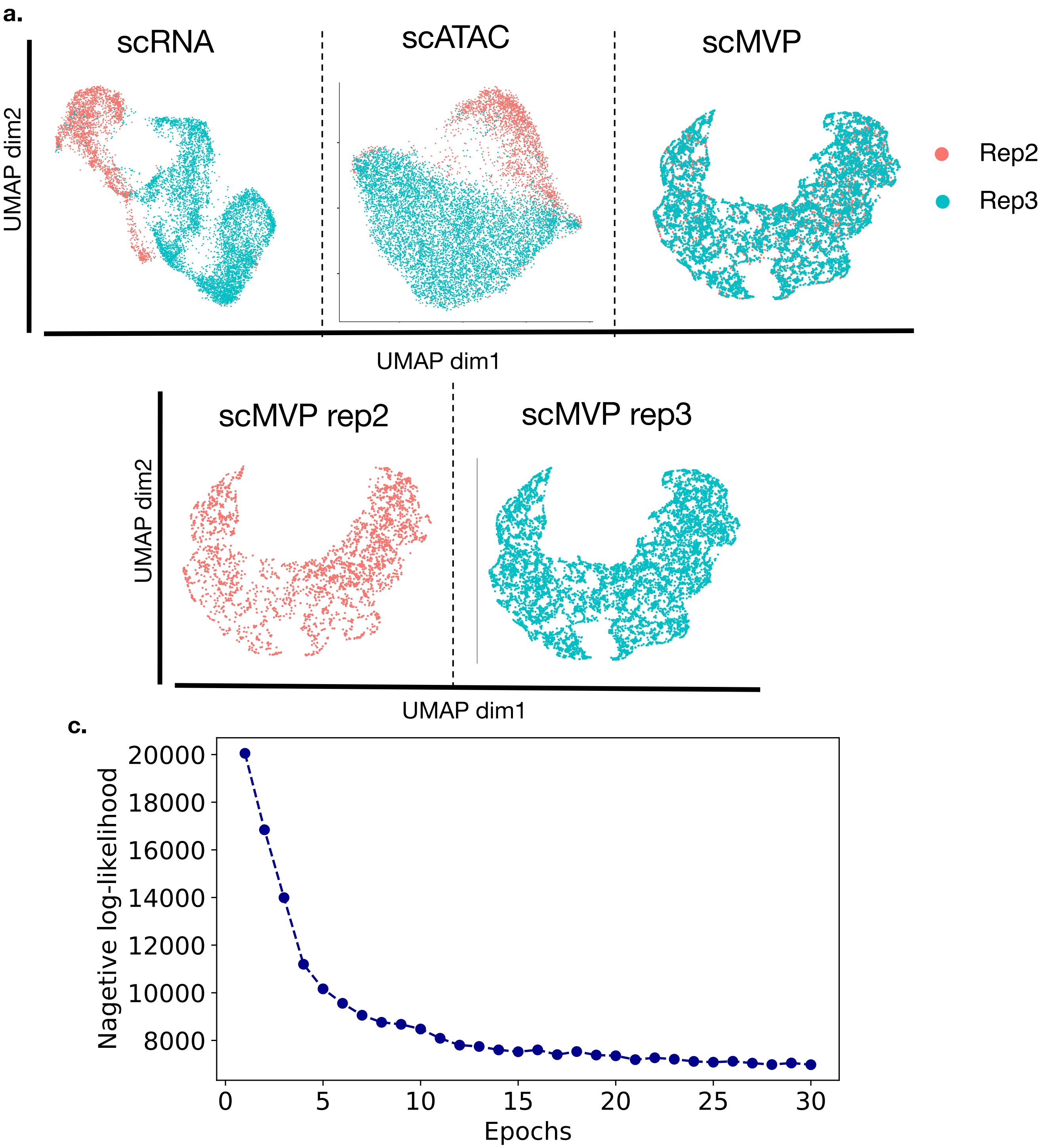

Fig.S1 scMVP model evaluation. **a.** UMAP visualization of SHARE-seq GM12878 cell line dataset of replicate 2 and 3 with 2,973 cells and 8,803 cells. **b.** Negative log-likelihood of ELBO loss in each epoch of training on the SHARE-seq dataset. This plot shows the fast convergence characteristic during training of scMVP model.

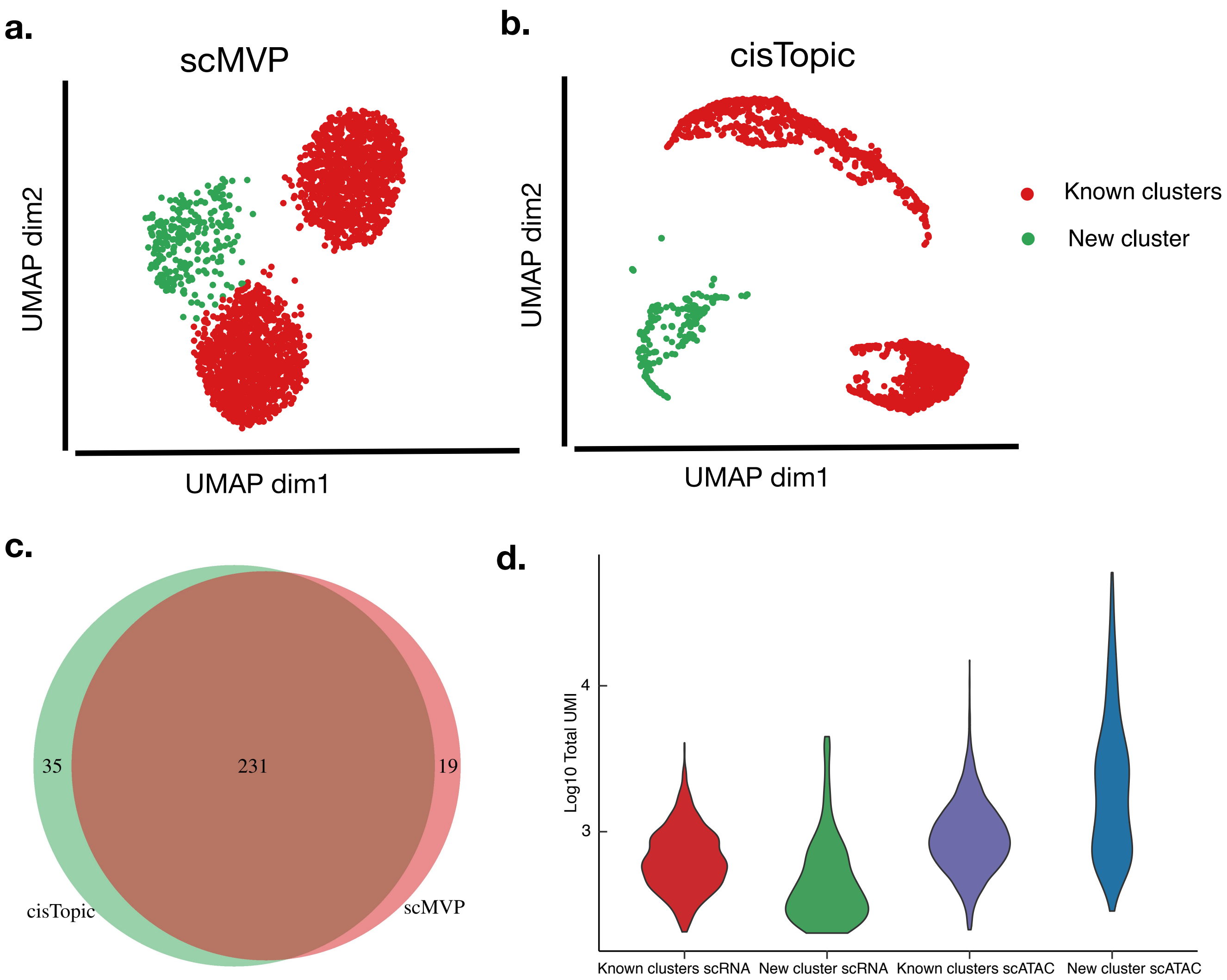

Fig.S2 Identification of Paired-seq new cell cluster. **a-b.** UMAP visualization Paired-seq dataset of scMVP and cisTopic output, colored by known clusters and new cluster. **c.** Overlap analysis of cells in new cell cluster in scMVP (gene cells in Fig. S4a) and cisTopic (gene cells in Fig. S4b) output. **d.** Violin plot of RNA and ATAC expression for Paired-seq cell line dataset in cells of known clusters and new cluster.

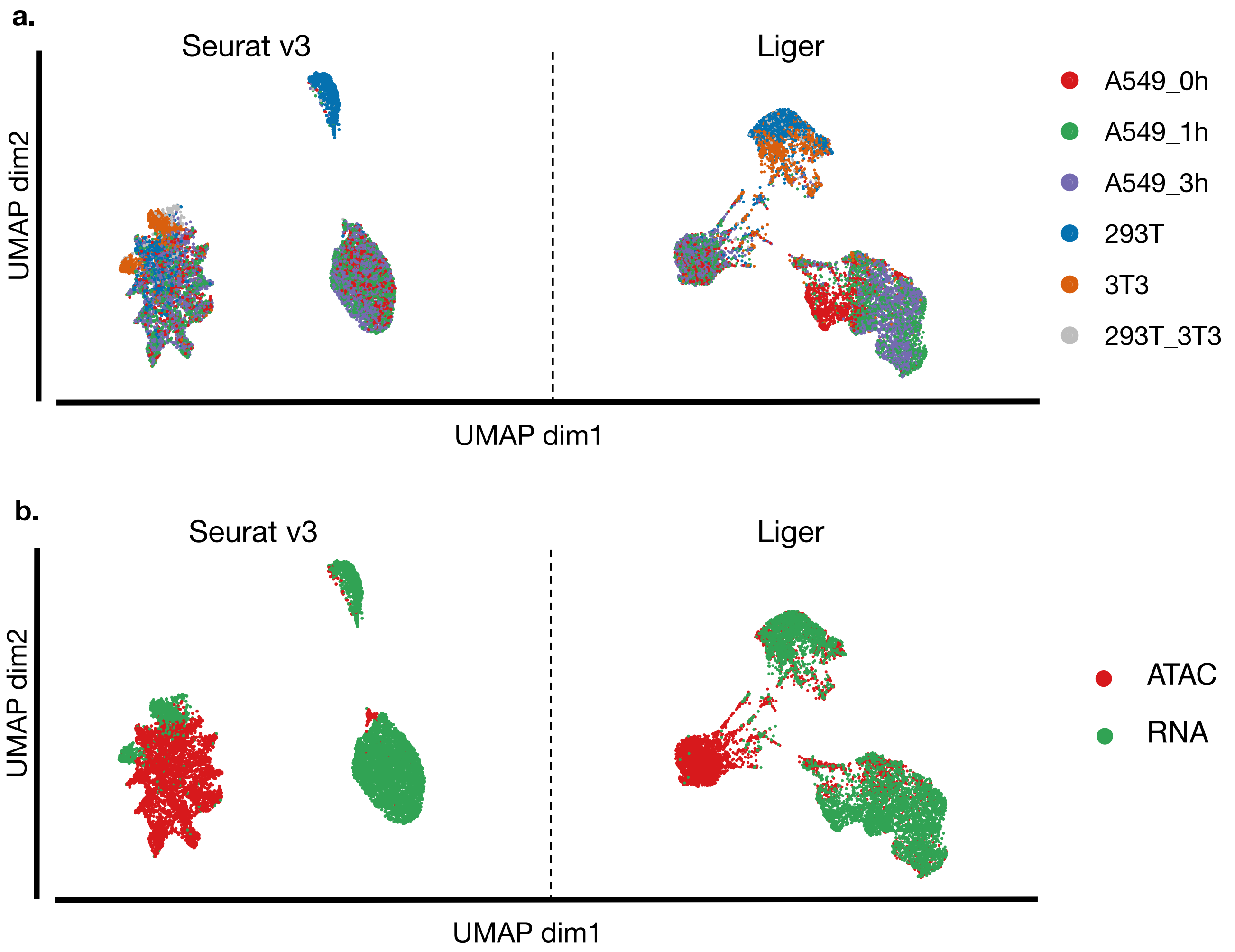

Fig.S3 UMAP visualization of Seurat v3 and Liger integration output of sci-CAR cell line dataset, colored by **a.** cell type and **b.** source of cells.

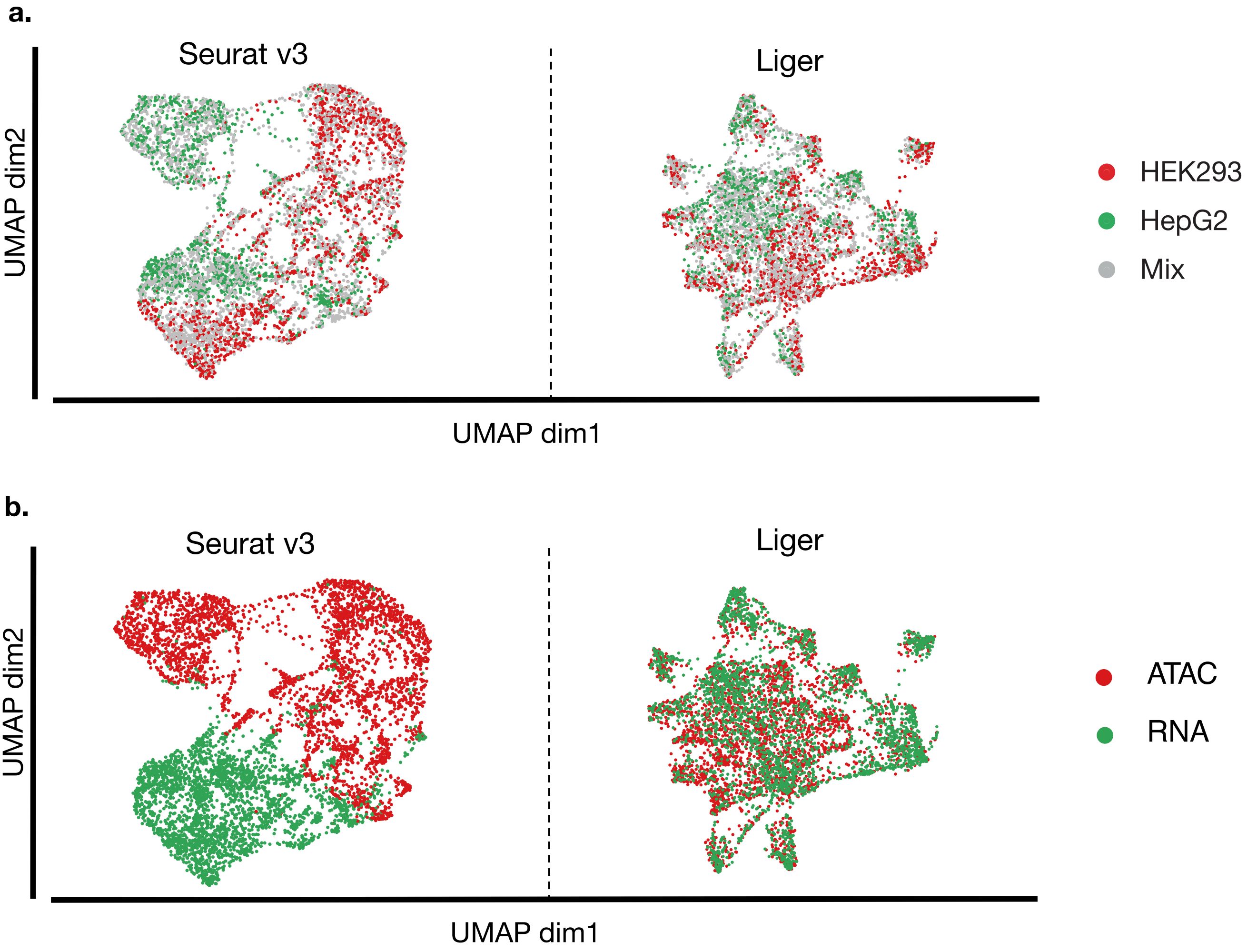

Fig.S4 UMAP visualization of Seurat v3 and Liger integration output of Paired-seq cell line dataset, colored by **a.** cell type and **b.** source of cells.

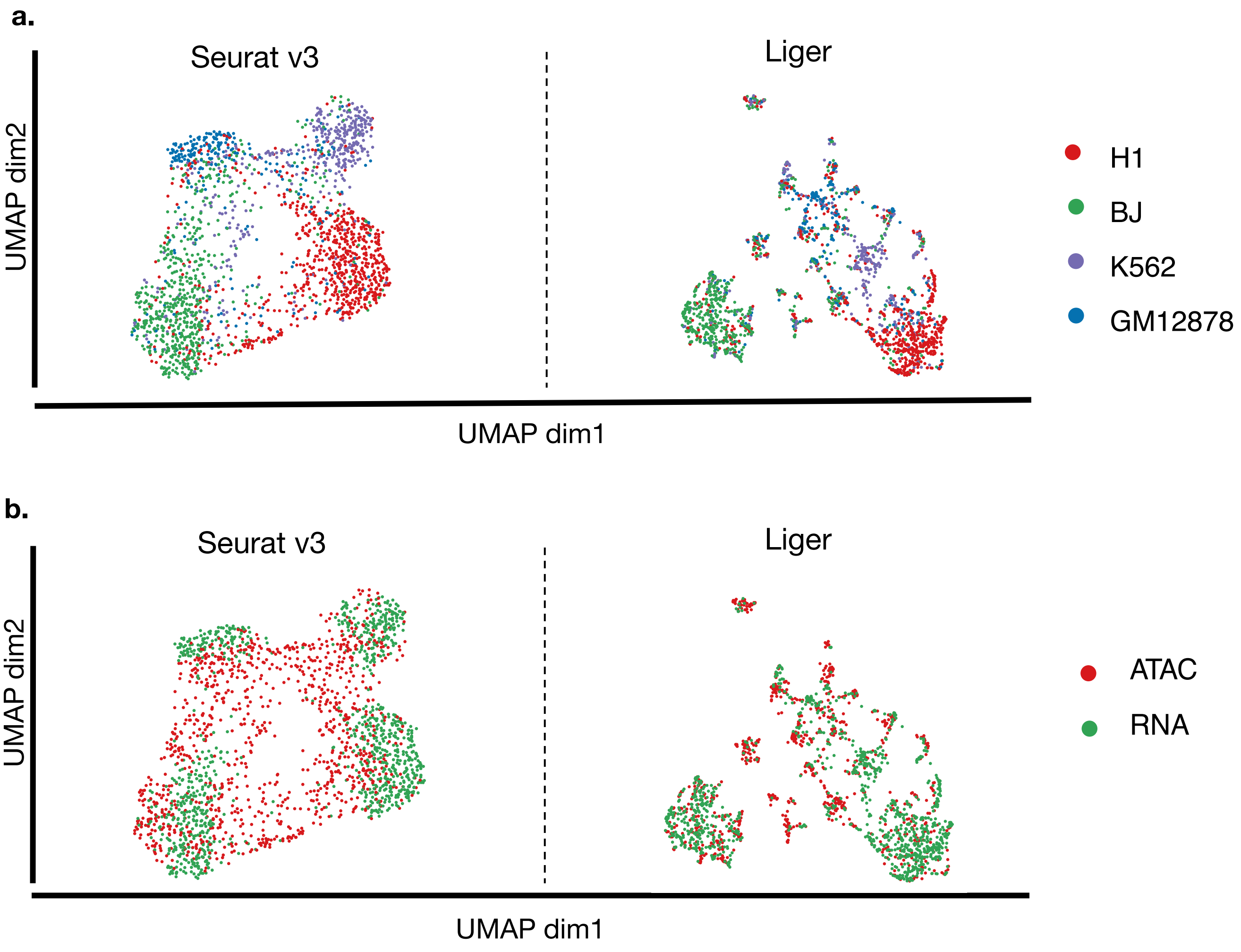

Fig.S5 UMAP visualization of Seurat v3 and Liger integration output of SNARE-seq cell line dataset, colored by **a.** cell type and **b.** source of cells.

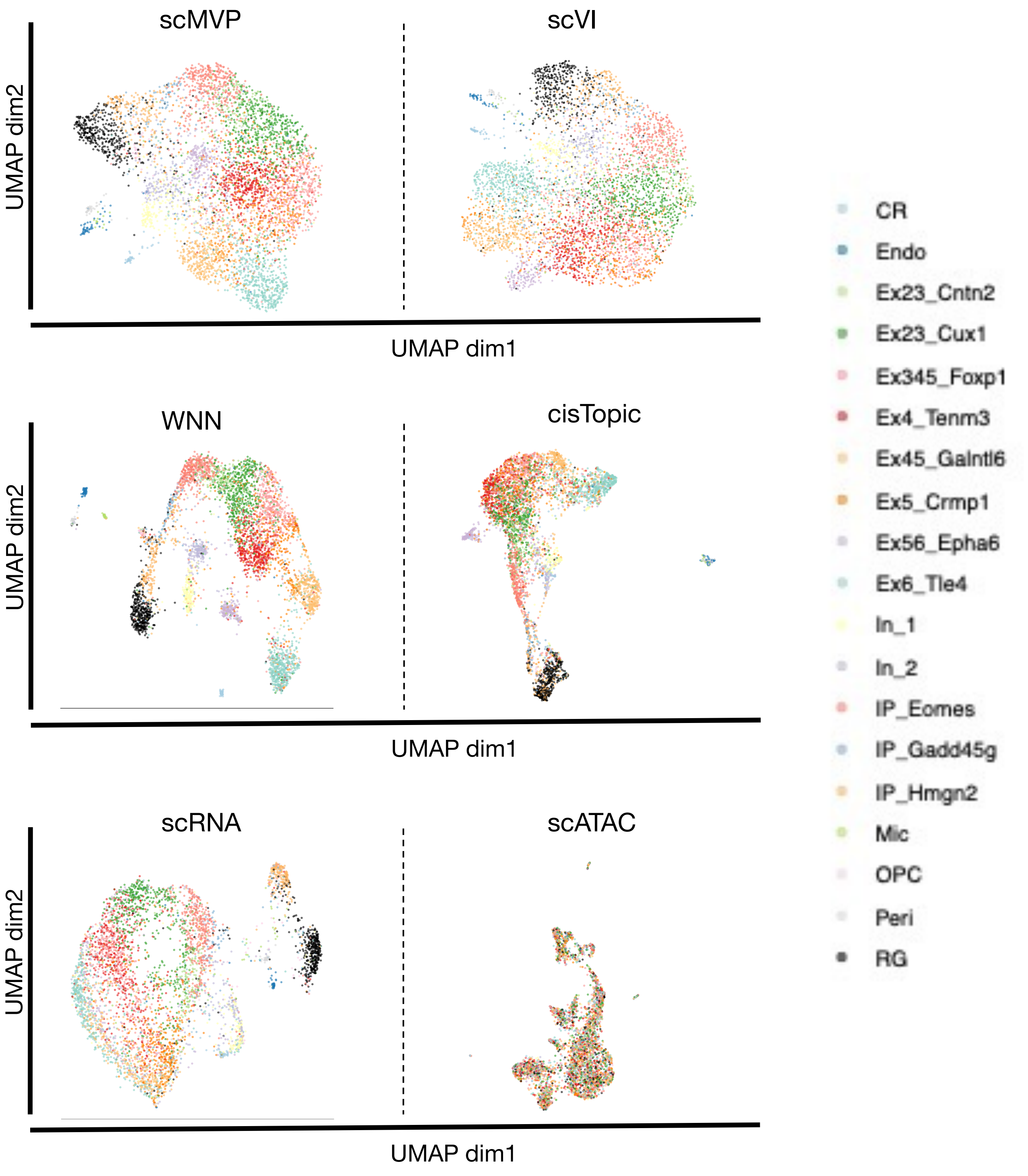

Fig.S6 UMAP visualization of scMVP, scVI, cisTopic, WNN by Seurat v4 , Monocle3 scRNA and scATAC on the SNARE p0 dataset

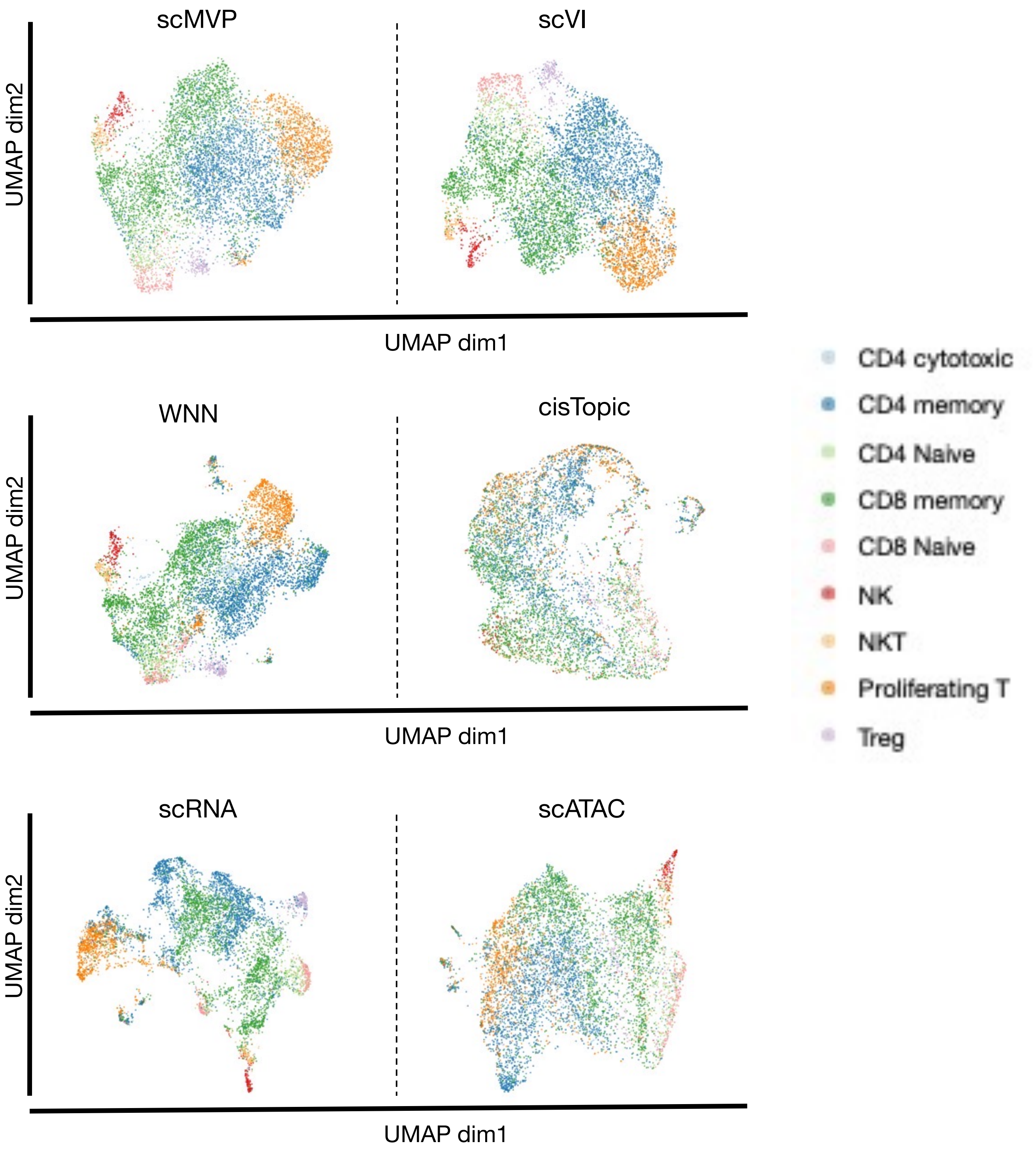

Fig.S7 UMAP visualization of scMVP, scVI, cisTopic, WNN by Seurat v4, Monocle3 scRNA and scATAC on T cell subtypes of the 10x genomics lymph node dataset

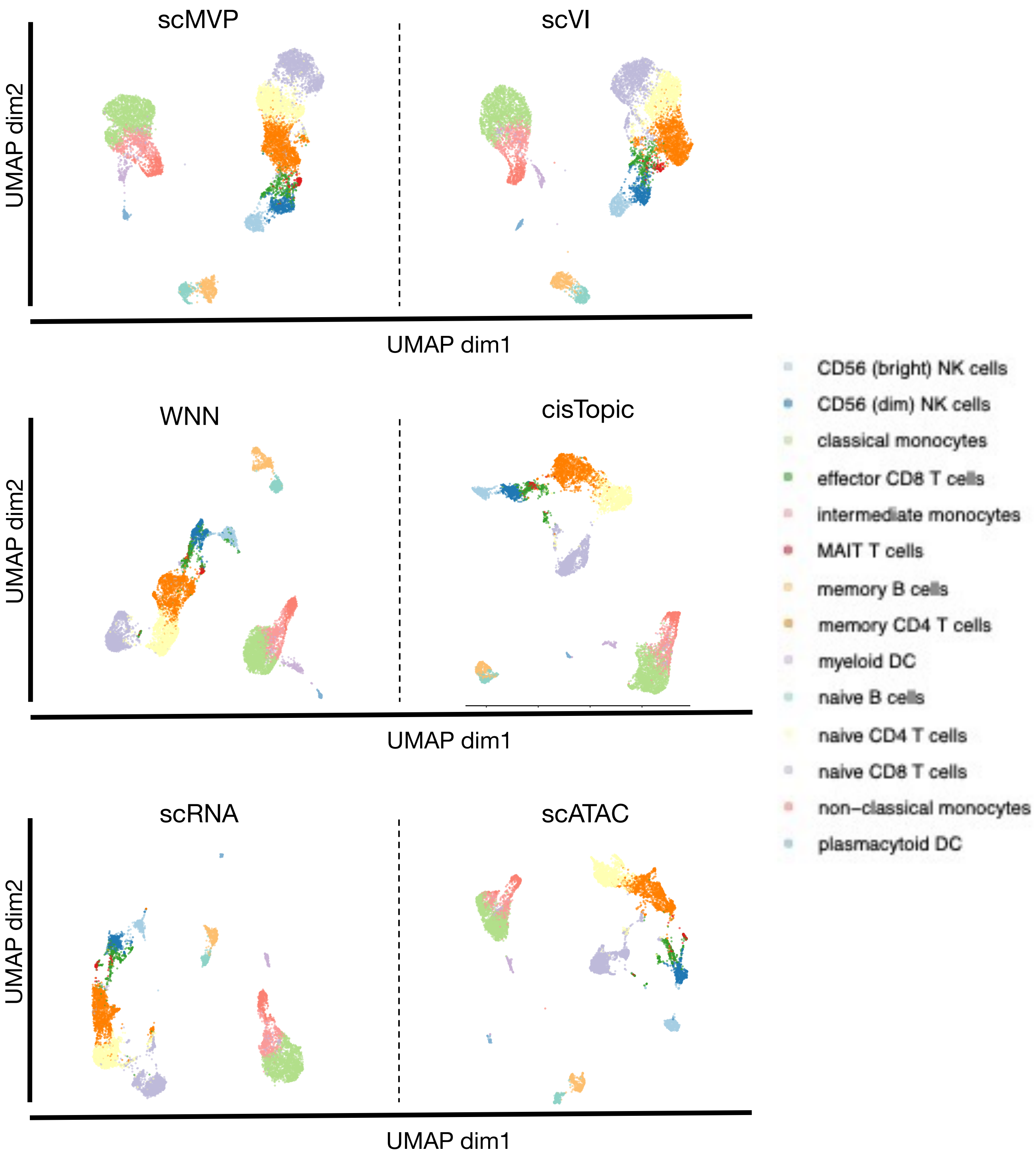

Fig.S8 UMAP visualization of scMVP, scVI, cisTopic, WNN by Seurat v4, Monocle3 scRNA and scATAC on the 10x genomics PBMC dataset

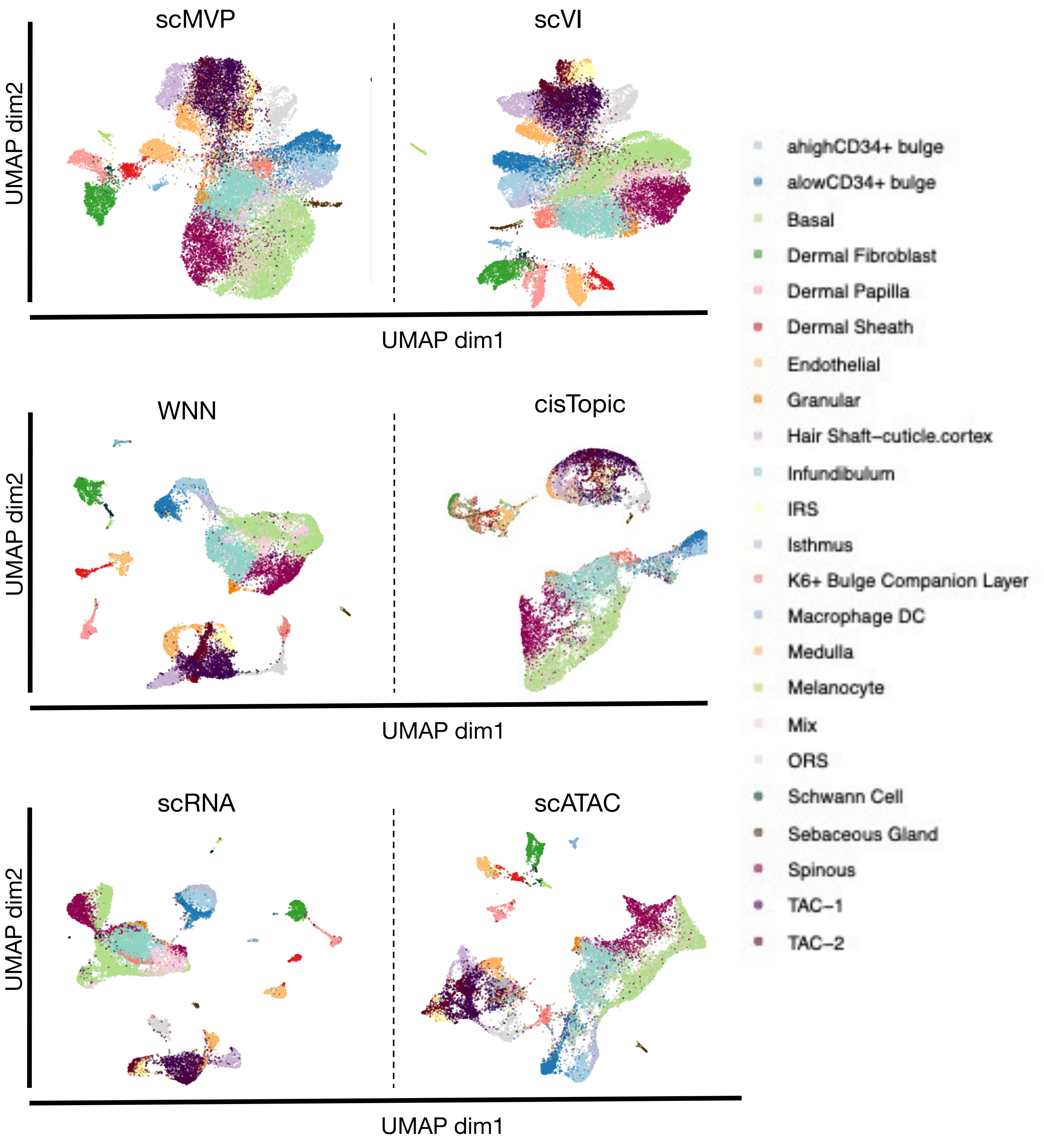

Fig.S9 UMAP visualization of scMVP, scVI, cisTopic, WNN by Seurat v4, Monocle3 scRNA and scATAC on the SHARE-seq mouse skin dataset

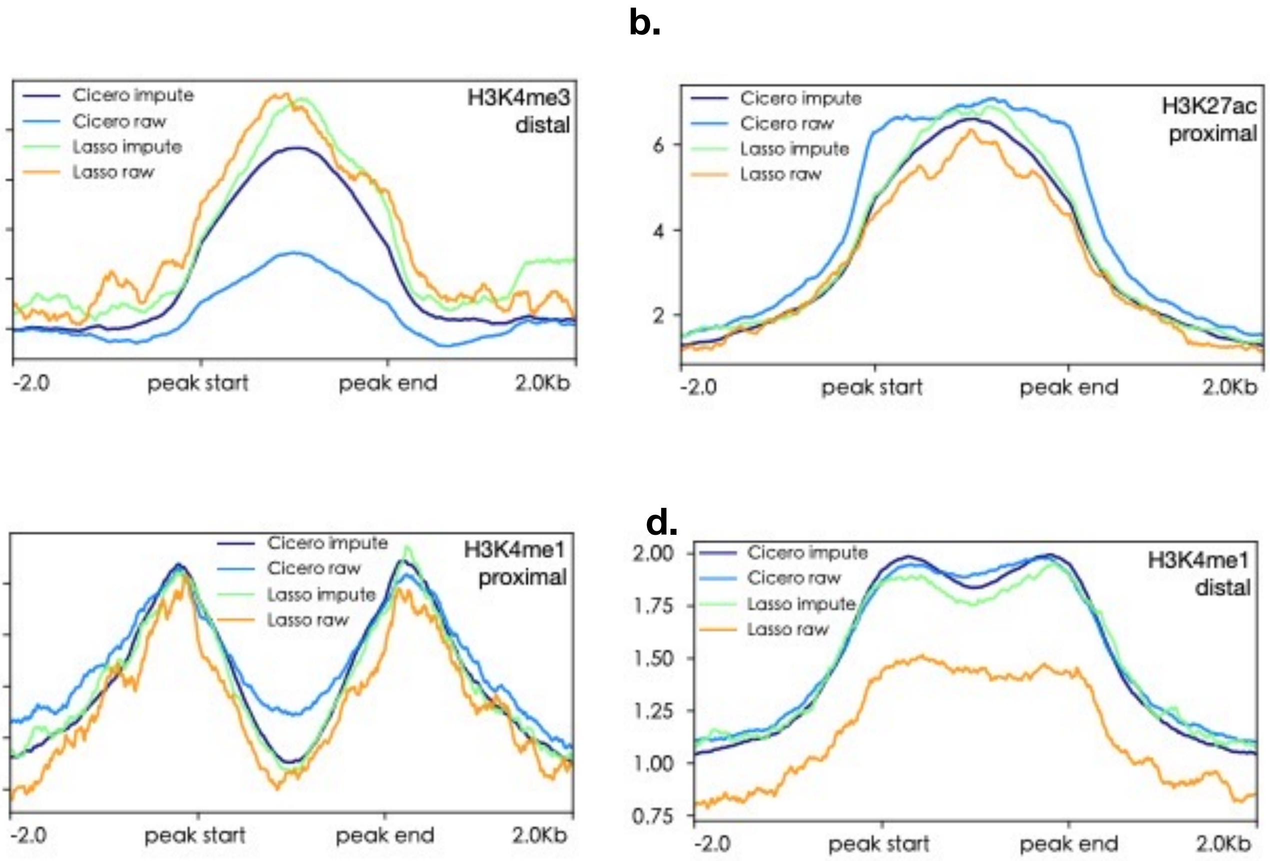

Fig.S10 The aggregate profile of mouse forebrain P0 ChIP-seq signal in candidate cis-regulatory peaks. This figure corresponds to Figure 4e and 4f, but for **a.** H3K4me3 signal (ENCSR094TTT) in distal peaks, **b.** H3K27ac signal (ENCSR094TTT) in proximal, **c.** H3K4me1 signal (ENCSR465PLB) in proximal peaks and **d.** H3K4me1 signal in distal peaks.

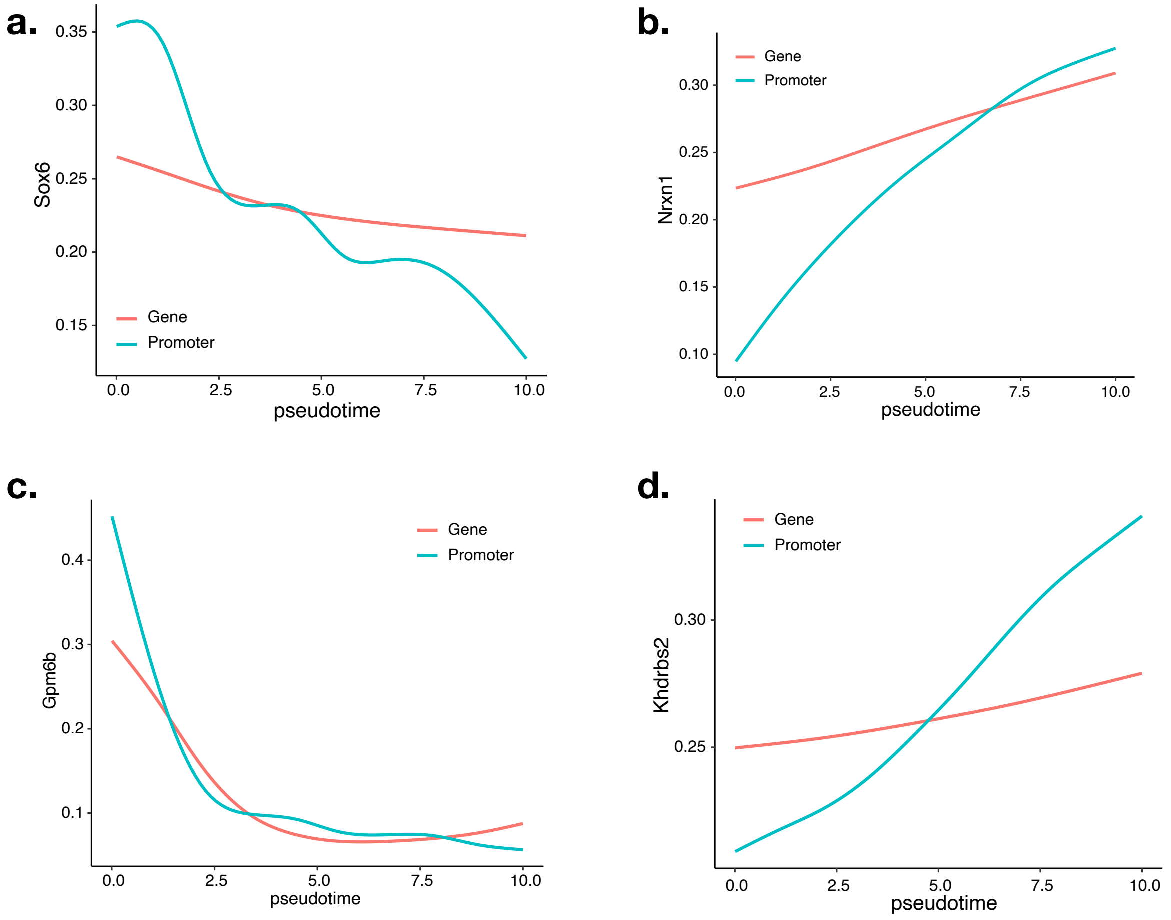

Fig.S11 Relative gene and promoter expression changes of a. Sox6 , b. Nrxn1, c. Gpm6b and d. Khdrbs2 across trajectory pseudotime in Fig. 5b.

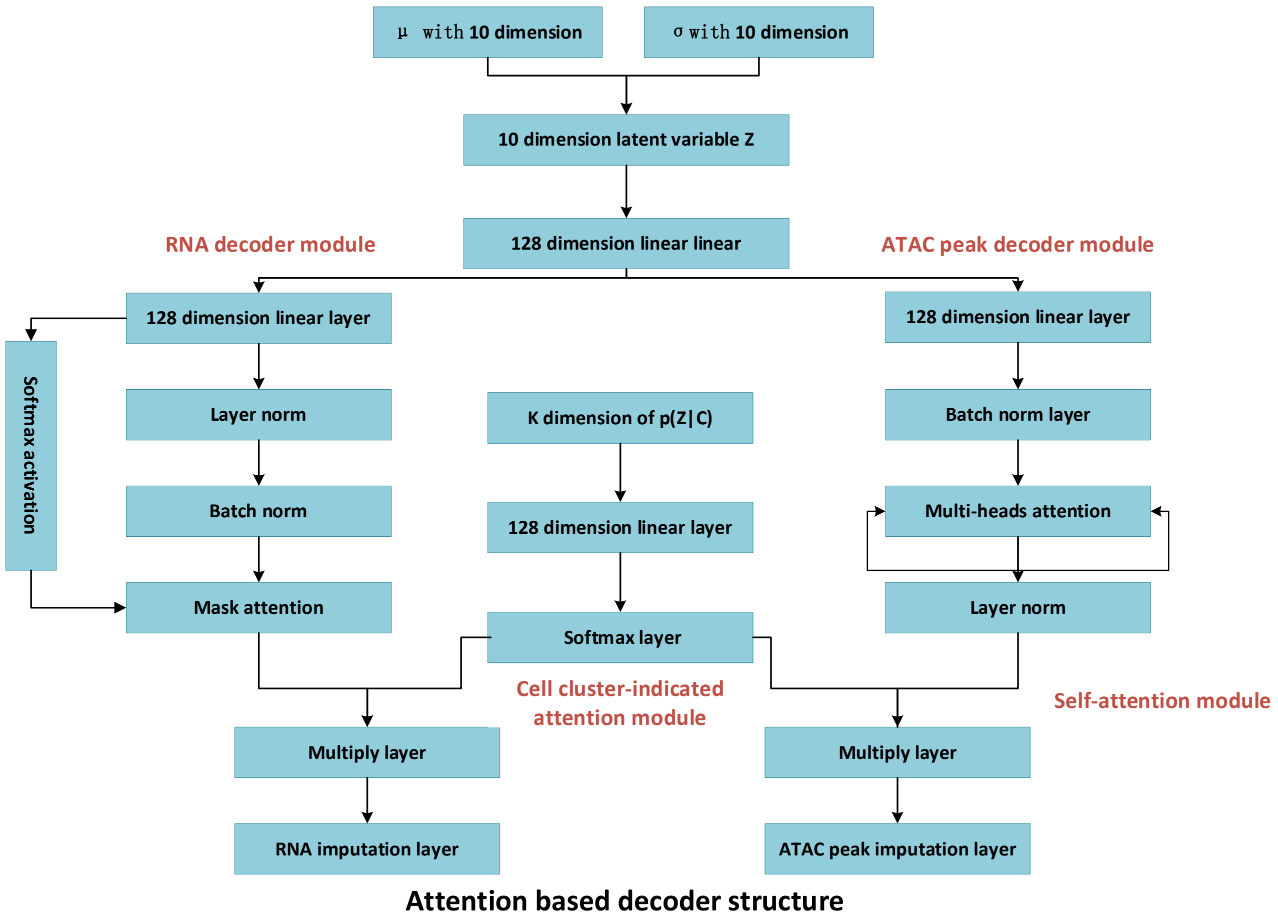

Fig. S12 Schematic diagram of scMVP decoder network**.**

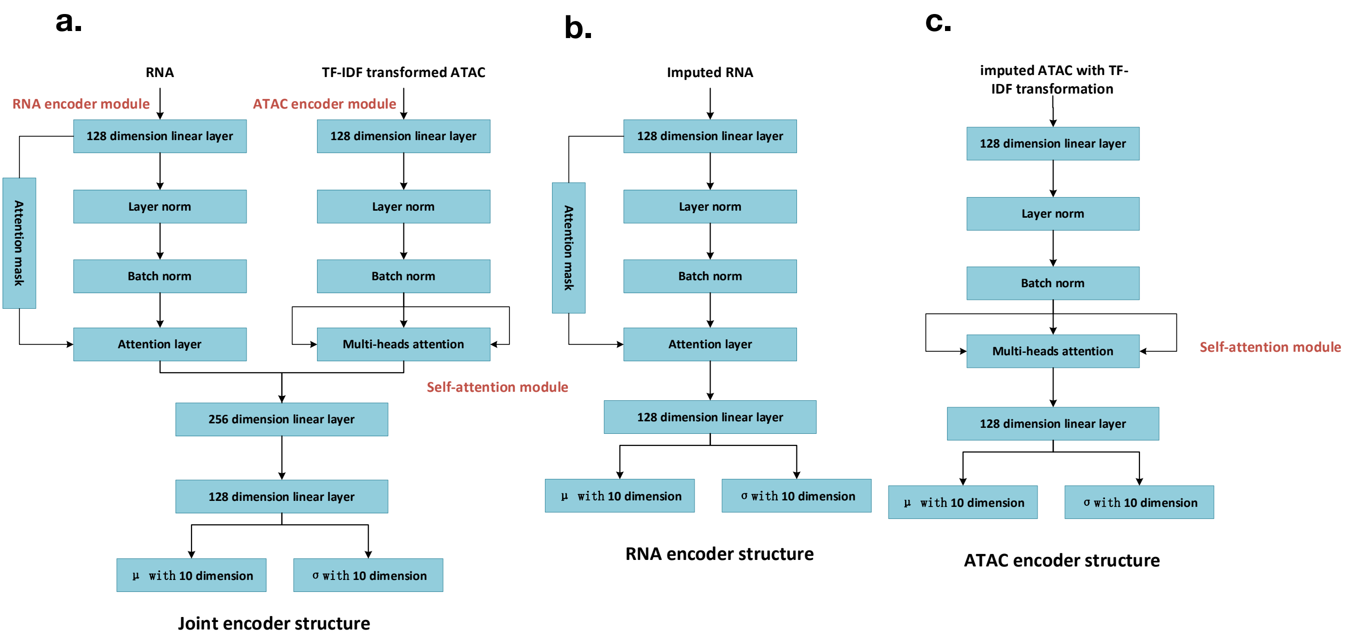

Fig. S13 Schematic diagram of scMVP encoder network**. a.** Joint encoder structure using scRNA and scATAC data as input, **b.** single encoder structure using scRNA as input, **c.** single encoder structure using scATAC as input.

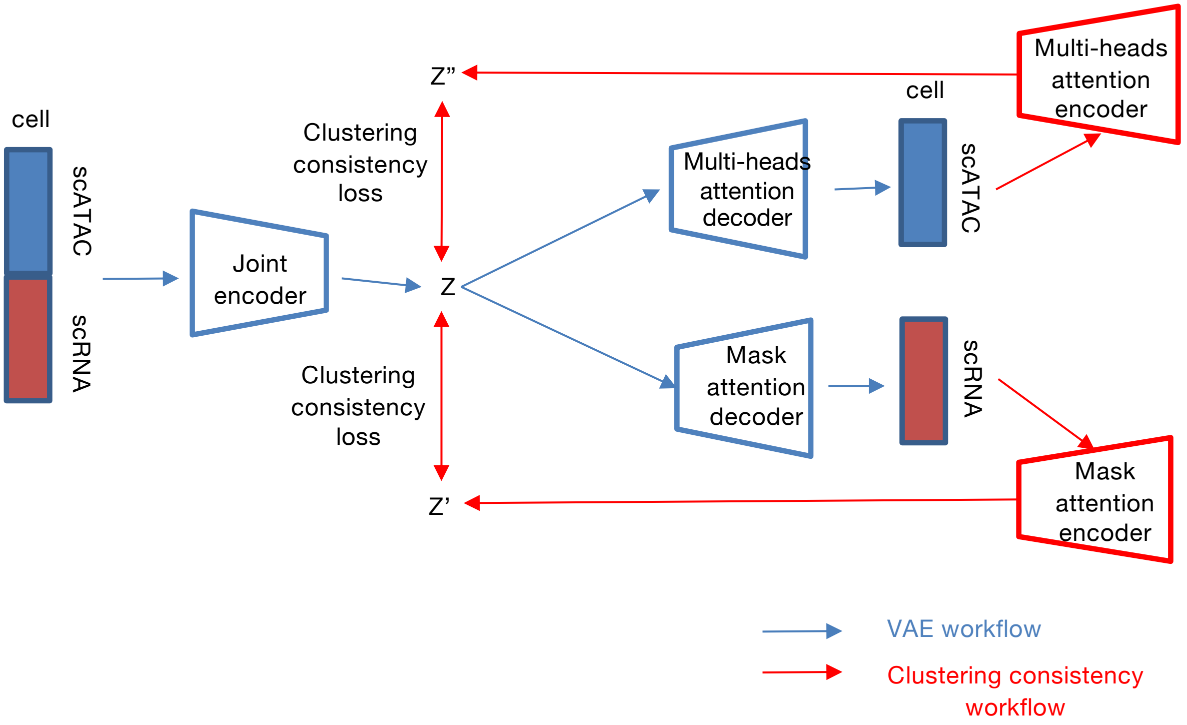

Fig. S14. A simplified diagram of scMVP model. The blue arrows indicate the workflow of multi-VAE based embedding framework, including multi-heads self-attention-based transformer module and mask attention-based module. The joint encoder represents both the multi-heads attention-based transformer module for raw scATAC profile and mask attention-based module for raw scRNA profile. The red arrows indicate the process of cycling clustering and consistency embedding from the imputed scRNA and scATAC.
